# WORKWELL process evaluation: qualitative data analyses of the participant interviews at 12- and 36-month follow-ups

**DOI:** 10.1093/rap/rkaf034

**Published:** 2025-03-14

**Authors:** Simone Battista, Jennifer Parker, Angela Ching, June Culley, Sarah Long, Alison Heard, Alison Hammond, Kathryn Radford, Paula Holland, Terence O’Neill, Karen Walker-Bone, Yeliz Prior

**Affiliations:** School of Health and Society, Centre for Human Movement and Rehabilitation, University of Salford, Salford, UK; School of Health and Society, Centre for Human Movement and Rehabilitation, University of Salford, Salford, UK; School of Health and Society, Centre for Human Movement and Rehabilitation, University of Salford, Salford, UK; Patient Research Partner, East Midlands, UK; Patient Research Partner, East Midlands, UK; Patient Research Partner, East Midlands, UK; School of Health and Society, Centre for Human Movement and Rehabilitation, University of Salford, Salford, UK; Centre for Rehabilitation and Ageing Research, University of Nottingham and Nottingham Biomedical Research Centre, Nottingham, UK; Division of Health Research, Lancaster University, Lancaster, UK; Centre for Epidemiology Versus Arthritis, The University of Manchester, Manchester, UK; NIHR Manchester Biomedical Research Centre, Manchester Academic Health Sciences Centre, Manchester University Foundation NHS Trust, Manchester, UK; Monash Centre for Occupational and Environmental Health, Monash University, Melbourne, UK; Versus Arthritis and MRC Centre for Musculoskeletal Health and Work, University of Southampton, Southampton, UK; School of Health and Society, Centre for Human Movement and Rehabilitation, University of Salford, Salford, UK

**Keywords:** job security, rehabilitation, vocational, occupational therapy, working conditions, occupational stress, qualitative research, outcome and process assessment, health care, intervention implementation science

## Abstract

**Objectives:**

This study aimed to qualitatively examine the delivery of the WORKWELL trial, a job retention vocational rehabilitation (JRVR) programme designed to help individuals with inflammatory arthritis (IA) maintain employment. A qualitative process evaluation used the Normalization Process Theory (NPT) to understand participant experiences and identify factors influencing implementation and outcomes.

**Methods:**

Data were collected via one-to-one telephone interviews with trial participants at 12 and 36 months. An inductive reflexive thematic analysis was followed by a deductive analysis based on NPT’s four constructs (coherence, cognitive participation, collective action and reflexive monitoring).

**Results:**

Sixty-two participants (mean age 51.0; 82.3% female) were interviewed, most diagnosed with RA (75.8%). Four secondary themes were generated under NPT constructs. For ‘Coherence’, themes included ‘Exploring the Purpose and Impact of Taking Part in WORKWELL’ and ‘Questionnaires as Instrument for Reflection’. In ‘Cognitive Participation’, the theme was ‘Commitment and Investment to WORKWELL’. For ‘Collective Action’, we identified ‘Key Actions for Successful WORKWELL’, and under ‘Reflexive Monitoring’, the theme was ‘Suggestions for Improving WORKWELL’. These themes reflected participants’ mixed feelings about the intervention, finding value in the intervention but highlighting the need for more tailored, timely and relevant content. Workplace support was crucial but often insufficient. Follow-up calls from researchers to ensure questionnaire completion were seen as a way to reflect and monitor their conditions. The pandemic’s impact on work environments also influenced outcomes.

**Conclusion:**

Findings suggest that WORKWELL provided work support for participants, though its impact could be enhanced through greater customization, early intervention and stronger workplace engagement.

**Trial registration:**

ClinicalTrials.gov NCT03942783. Registered on 8 May 2019. ISRCTN Registry ISRCTN61762297. Registered on 13 May 2019. Retrospectively registered.

Key messages

- Participants valued the provided support but emphasized the need for tailored content and timing.
- Participants viewed study calls and questionnaire completion as a helpful health-monitoring tool.
- Workplace engagement is a key factor in maintaining employment, though employer buy-in needs to be improved.

## Introduction

Work is important to individuals, providing societal status, purpose, self-esteem, financial independence and better physical and mental health [1, 2]. Individuals with inflammatory arthritis (IA) [e.g. RA, axial spondylarthritis (AxSpa) and PsA] often encounter challenges in the workplace, such as work instability, presenteeism (loss of productivity) and absenteeism (sick leave), which can lead to work disability (i.e. job loss) [3]. However, people with IA highlighted the importance of remaining employed [4].

Job retention vocational rehabilitation (JRVR) supports employed individuals facing challenges in maintaining employment [5]. The European Agency for Safety and Health at Work (EU-OSHA) identified key factors for successful rehabilitation and return-to-work systems, highlighting comprehensive frameworks in countries like Germany, Denmark, Austria, the Netherlands, Norway and Sweden, with the UK not having similar comprehensive programmes, above all in the rheumatic field [6]. Hence, the WORKWELL trial was established in the UK to evaluate the clinical and cost-effectiveness of JRVR for employed people with IA experiencing work-related issues [3, 7]. This intervention is based on a multicentre randomised controlled trial (RCT) delivered by trained National Health Service (NHS) occupational therapists (OTs) and built upon successful JRVR trials [8–10]. The WORKWELL JRVR intervention begins with a self-help written information pack, including practical work support and details on the Equality Act [3, 7]. For the intervention group only, the programme follows with a comprehensive work interview with OTs based on the Work Experience Survey-Rheumatic Conditions (WES-RC) to identify work barriers, prioritize three key work-related problems and create an individualized JRVR plan [3, 7]. Up to three additional treatment sessions and a follow-up phone review are provided to assess progress and job accommodation implementation [3, 7].

The UK Medical Research Council framework guides the systematic approach to process evaluations in trials involving complex interventions, stressing the importance of clear intervention theory and targeted process questions [11]. Normalization Process Theory (NPT) aids in understanding how patients, healthcare professionals and other stakeholders integrate new practices into their personal and professional lives to understand factors influencing implementation [11, 12]. Therefore, we conducted a qualitative interview study nested within the RCT, using the NPT framework to understand the factors influencing the implementation of the WORKWELL JRVR intervention.

## Methods

### Study design

This qualitative interview study uses the NPT framework to interpret the WORKWELL intervention and its implementation. We explored participants’ perspectives at 12 and 36 months. A patient and public involvement (PPI) group was established (see ‘Patient and public involvement’ section below). This study is reported following the Consolidated Criteria for Reporting Qualitative Research (COREQ) [13]. Ethical approval was received from the Health Research Authority West Midlands—Solihull Research Ethics Committee (18/WM/0327) and the University of Salford Research, Enterprise, and Engagement Ethical Approval Panel (HSR1819-010). WORKWELL study protocols have been previously published [3, 7, 14].

### Participants

Individuals in control (usual care + self-help written information pack) and intervention (usual care + self-help written information pack + WORKWELL JRVR) groups who had completed the 12- and 36-month follow-ups were contacted through post or email with an interview invitation letter, participant information sheet and consent form. To be eligible, participants needed to be aged >18 years, be diagnosed with IA by a Rheumatology Consultant, work at least 15 h per week in paid employment, score ≥10 on the RA—Work Instability Scale (RA-WIS) (moderate to high risk of work instability), be able to attend WORKWELL appointments, understand English and provide informed consent. Individuals were excluded if they were on extended sick leave (>4 weeks), planning to retire within 12 months, moving out of the area within 4 months, already receiving or awaiting other JRVR interventions, or employed in the armed forces, which have their JRVR services [7]. The original study protocol was designed to interview only participants from the intervention group. However, the PPI group recommended expanding the scope to include control group participants, which could provide valuable insights into those who only received the resource pack. Purposive sampling [15] was adopted to assemble a diverse study cohort, considering gender, job skill levels [16], work status, ethnicity and the period of the study within which participants were recruited to ensure the inclusion of those whose participation was interrupted by the COVID-19 pandemic [14]. Subsequently, participants were reached via telephone or email a week later to explain the study’s aim and confirm their willingness to participate.

The sample size was determined using the concept of ‘information power’ rather than the commonly used but methodologically inappropriate ‘data saturation’ for reflexive thematic analysis (RTA) [17]. Given the researchers’ expertise in qualitative research and IA, the solid theoretical foundations of our study, the specificity of our research question, and the purposeful selection process, an estimate of 15–20 participants per group (researchers’ interviews at 12 months, PPI’s interviews at 12 months and researchers’ interviews at 36 months) was considered necessary [18].

### Data collection

Semi-structured interview guides were developed informed by NPT with the study team of researchers, rheumatology health professionals and patient research partners (Supplementary Table S1, available at *Rheumatology Advances in Practice* online). At 12 months, the topic guides aimed to prompt participants to reflect on their experiences of the WORKWELL trial. Additional questions were later included to explore the impact of the COVID-19 outbreak [14]. At the 36-month follow-up, the interview guide focused on understanding the long-term effect of the WORKWELL trial. All interviews were conducted by telephone at a mutually convenient date and time for the participants. The PPI group members (J.C., S.L., A.He.) interviewed participants from both groups at 12 months using an interview guide they developed (Supplementary Table S1, available at *Rheumatology Advances in Practice* online). A.C. interviewed the intervention and control groups at the 12-month follow-up. At 36 months, participants from the control group were interviewed by Y.P., and J.P. interviewed the participants in the intervention group. The researchers interviewed all participants alone, and they did not know the interviewees before approaching the study.

### Data analysis

The interviews were audio-recorded and transcribed *verbatim* with names replaced by pseudonyms for people interviewed by the researchers and codes for those interviewed by the PPI members. PPI members preferred using codes over pseudonyms. Transcripts were not returned to participants but were checked for accuracy. The transcripts were inductively analysed following the six steps (Table 1) of the RTA [19, 20], a constructionist paradigm, an experiential orientation and semantic coding [21]. RTA is an interpretive approach to qualitative data analysis that facilitates the identification and analysis of patterns or themes within a data set [19, 20]. We employed this approach to identify patterns of meaning related to the factors that undermine the implementation of WORKWELL. RTA was chosen for its flexibility and adaptability to complex experiences, making it well suited for our study [19, 20]. Themes previously coded were grouped under the various NPT constructs and components through a theory-driven deductive analysis. NPT comprises four key constructs—coherence (making sense of the intervention), cognitive participation (engaging and committing to the intervention), collective action (implementing and executing the intervention) and reflexive monitoring (evaluating and adjusting the intervention) [12].

**Table 1.** Six steps of the RTA

Phases	Process	Authors’ involvement	Authors’ actions
1. Data familiarization	S.B., J.P., A.C., Y.P. and the PPI members read and reread several times the transcriptions of the interviews. This process is fundamental to getting in contact with the data and taking notes of any insights.	All authors engaged in this phase, and they met to reflect upon their first insights.	- Document theoretical and reflective thoughts: documented field notes (‘Memos’ and diary) on the interviews to promote reflexivity. - Keep records of all data field notes, transcripts and reflexive diary. - Prolong engagement with data and triangulate different data collection modes to increase the probability that the research findings and interpretations will be found credible.
2. Coding	In this phase, the researchers systematically coded the data through an open, evolving and organic process.	S.B., A.C. and the PPI members coded the data for interviews. Y.P. oversaw the PPI analysis. The coding was shared with the whole group. They adopted semantic data coding.	- Peer debriefing: memos were shared during research meetings for reflexive thoughts. - Audit trail of code generation: S.B. coded data through the entire data set to identify interesting aspects in the data items that may form the basis of themes across the data set. - Documentation of all team meetings and peer debriefings to help researchers examine how their thoughts and ideas evolve as they engage more deeply with the data.
3. Generating initial themes	The researchers generated initial themes from the codes, clustering similar or related codes.	S.B., A.C. and the PPI members generated initial themes separately, clustering similar codes together. J.P. and Y.P. oversaw the whole process.	- Diagramming to make sense of theme connections: S.B., A.C. and the PPI members generated initial themes.
4. Reviewing and refining themes	The researcher reviewed the initial themes, reworking or discarding some until finding a final set of themes fitting the data.	All authors reviewed the coding and initial themes to generate the themes that fit the data the most.	The research team frequently met to refine the themes and clearly show how each theme was derived from the data.
5. Defining and naming themes	The ‘story’ of each theme is developed by finalizing theme names and their definition.	All authors finalized the final themes and definitions to set the basis of the written report.	- Peer debriefing and team consensus on themes: the research team met until the final themes were reached. - Documentation of theme naming.
6. Producing the report	The authors produced the final report and refined it if necessary.	S.B., A.C., J.P., Y.P. and the PPI members selected the illustrative quotations from the interviews, and all authors reviewed and agreed. S.B. and Y.P. led the writing of the paper, and all authors participated in this phase.	- Producing the report using direct quotes from participants. - Report on reasons for theoretical, methodological and analytical choices throughout the entire study.

S.B. is a physiotherapist, PhD and research fellow in physiotherapy and identifies as male. J.P. is a clinical trial manager, PhD and identifies as female. A.C. is a clinical trial manager, PhD and research fellow and identifies as female. Y.P. is an occupational therapist, PhD and professor of clinical rehabilitation and identified herself as female. All researchers are interested in RMD and are experienced in conducting qualitative research.

RTA: reflexive thematic analysis; PPI: patient and public involvement; RMD: rheumatic and musculoskeletal diseases.

S.B., A.C. and Y.P. analyzed the qualitative interviews collected by the researchers at the 12-month follow-up. S.B., Y.P. and PPI members analysed the qualitative interview data collected by the PPI group. S.B., Y.P. and J.P. analysed the interviews at the 36-month follow-up. NVivo was adopted to analyse the transcripts. In RTA, the researchers embrace the understanding that researcher subjectivity is an inherent and valuable part of the analytic process rather than a source of bias [19, 20]. The diverse professional backgrounds of the research team enriched the analysis by bringing varied perspectives, fostering deeper interpretation and enhancing reflexive engagement with the data.

### Patient and public involvement (PPI)

Table 2 reports the PPI group’s participation using the short form of the Guidance for Reporting Involvement of Patients and the Public (GRIPP2) [22].

**Table 2.** Guidance for Reporting Involvement of Patients and the Public (GRIPP2) short form

GRIPP2 reporting item	Description
1. Aim	The primary aim of the PPI group in the study was to ensure a patient-centred approach by incorporating the perspectives, experiences and preferences of individuals with IA into the process evaluation of the WORKWELL Trial. The PPI group contributed to key elements, including the creation of the interview guides, undertaking a number of interviews and the interview analysis.
2. Methods	Three PPI members with IA contributed throughout the process evaluation of the trial. J.C. (lead PPI member) worked with the research team as a PPI member for a number of years on studies predating the WORKWELL trial. She identified S.L. and A.He. as additional members. They are all working or retired women with RA in the East Midlands area of the UK. With them, we conducted eight PPI meetings over 2 years, mostly online due to COVID-19. The outcomes of the meetings were reported to the TMG and TSC by J.C. The PPI group participated in the development of all interview topic guides. In addition, they developed the topic guide for PPIE-led interviews with participants that took place at 12 m between March 2021 and May 2022; they also performed and analysed this sub-group of interviews. This guide was reviewed by A.C. and Y.P. Y.P. trained the PPI members to analyse resulting qualitative data through RTA through eight online meetings over a 2-year period. They also participated in the interpretation of results and discussions on dissemination strategies for communicating trial findings to different stakeholders.
3. Study results (outcomes)	The PPI group successfully contributed to the trial’s process evaluation and interpretation of findings. Positive outcomes included: (1) the creation of a patient-centred interview guide; (2) the accepted proposal to interview individuals in the control group, which was not an initial aim of the study, positively influencing the results of our process evaluation; (3) providing clear guidance on communicating trial results to people with IA and their employers; and (4) collaborative involvement in the thematic analysis of patients’ interviews. Negative outcomes included challenges in holding in-person meetings due to COVID-19, which limited interaction among PPI members.
4. Discussion and conclusions (outcomes)	PPI had a significant influence on the study by ensuring that the perspectives of working individuals with IA were incorporated into the study design, evaluation and dissemination. The PPI group’s input enriched the trial’s relevance to real-world experiences. Positive effects included improving the accessibility of trial findings to patients and professionals. Negative effects were related to the logistical difficulties of maintaining active involvement during the pandemic. Nonetheless, the PPI group adapted well to virtual meetings.
5. Reflections/critical perspective	Reflecting on the experience, several aspects went well, such as the proposal to interview also the control group and the collaboration on thematic analysis and dissemination plans. Challenges included reduced opportunities for in-person interaction, which may have limited some deeper discussions.

PPI: patient and public involvement; IA: inflammatory arthritis; TMG: Trial Management Group; TSC: Trial Steering Committee; RTA: reflexive thematic analysis.

## Results

The final sample (Table 3) consisted of 62 individuals (age (s.d.): 51.0 (8.2), 51 F (82.3%), with a majority diagnosed with RA (*n* = 47, 75.8%), RA-WIS (s.d.): 15.7 (3.7) and the following skill levels: Level 1 (2 individuals, 3.2%), Level 2 (24 individuals, 38.7%), Level 3 (16 individuals, 25.8%), Level 4 (20 individuals, 32.3%). At the 12-month follow-up, 14 out of 249 participants (5.6%) declined to be contacted for an interview. All participants who consented to be contacted were invited for an interview. Only a few participants provided reasons for declining, with six citing lack of time. At the 36-month follow-up, participants were asked if they were willing to be contacted for an interview. Out of 180 participants, 90 (50%) agreed to be contacted, 73 (40.5%) declined, and 17 (9.5%) chose ‘prefer not to say’. All participants who consented to be contacted for an interview were invited via email. Reasons for declining were not collected. This is a fairly representative sample of the RCT population, which included 249 individuals (age (s.d.): 48.6 (9.9), 202 F (81.1%), with a majority diagnosed with RA (*n* = 159, 63.9%), RA-WIS (s.d.): 16.2 (4.4), Level 1 (16 individuals, 6.4%), Level 2 (100 individuals, 40.2%), Level 3 (56 individuals, 22.5%) and Level 4 (77 individuals, 30.9%).

**Table 3.** Descriptive data

Group *N* = 62	Age Mean (s.d.)	Gender *N* (%)	Diagnosis *N* (%)	RA-WIS Mean (s.d.)	Skill level *N* (%)
	12-month follow-up				
	*Researcher-led interviews*				
Intervention *N* = 17	49.6 (7.7)	F: 13 (76.5%) M: 4 (23.5%)	RA: 12 (70.6%) PsA: 5 (29.4%)	14.8 (3.1)	Level 2: 6 (35.3%) Level 3: 6 (35.3%) Level 4: 5 (29.4%)
Control *N* = 8	56.8 (5.9)	F: 8 (100%)	RA: 8 (100%)	17.4 (4.8)	Level 2: 3 (37.5%) Level 3: 1 (12.5%) Level 4: 4 (50.0%)
	*PPI-led interviews*				
Intervention *N* = 5	49.4 (15.2)	F: 4 (80%) M: 1 (20%)	RA: 5 (100%)	14.8 (2.6)	Level 1: 1 (20.0%) Level 2: 1 (20.0%) Level 3: 2 (40.0%) Level 4: 1 (20.0%)
Control *N* = 10	51.3 (8.8)	F: 8 (80.0%) M: 2 (20.0%)	RA: 6 (60.0%) PsA: 2 (20.0%) UIA: 1 (10.0%) EIA: 1 (10.0%)	15.3 (3.5)	Level 2: 4 (40.0%) Level 3: 2 (20%) Level 4: 4 (40%)
	36-month follow-up				
Intervention *N* = 10	50.4 (5.2)	F: 9 (90%) M: 10 (10%)	RA: 8 (80.0%) PsA: 2 (22.2%)	15.0 (3.5)	Level 2: 5 (50.0%) Level 3: 2 (20.0%) Level 4: 3 (30.0%)
Control *N* = 12	58.3 (7.1)	F: 9 (75.0%) M: 3 (25.0%)	RA: 8 (66.7) RA/PsA: 1 (8.3) PsA: 2 (16.7) UIA: 1 (8.3%)	17.1 (4.0)	Level 1: 1 (8.3%) Level 2: 5 (41.7%) Level 3: 3 (25.0%) Level 4: 3 (25.0%)

F: female; M: male; PPI: patient and public involvement; UIA: undifferentiated inflammatory arthritis; EIA: early inflammatory arthritis; RA-WIS: RA—Work Instability Scale.

**Figure 1.**
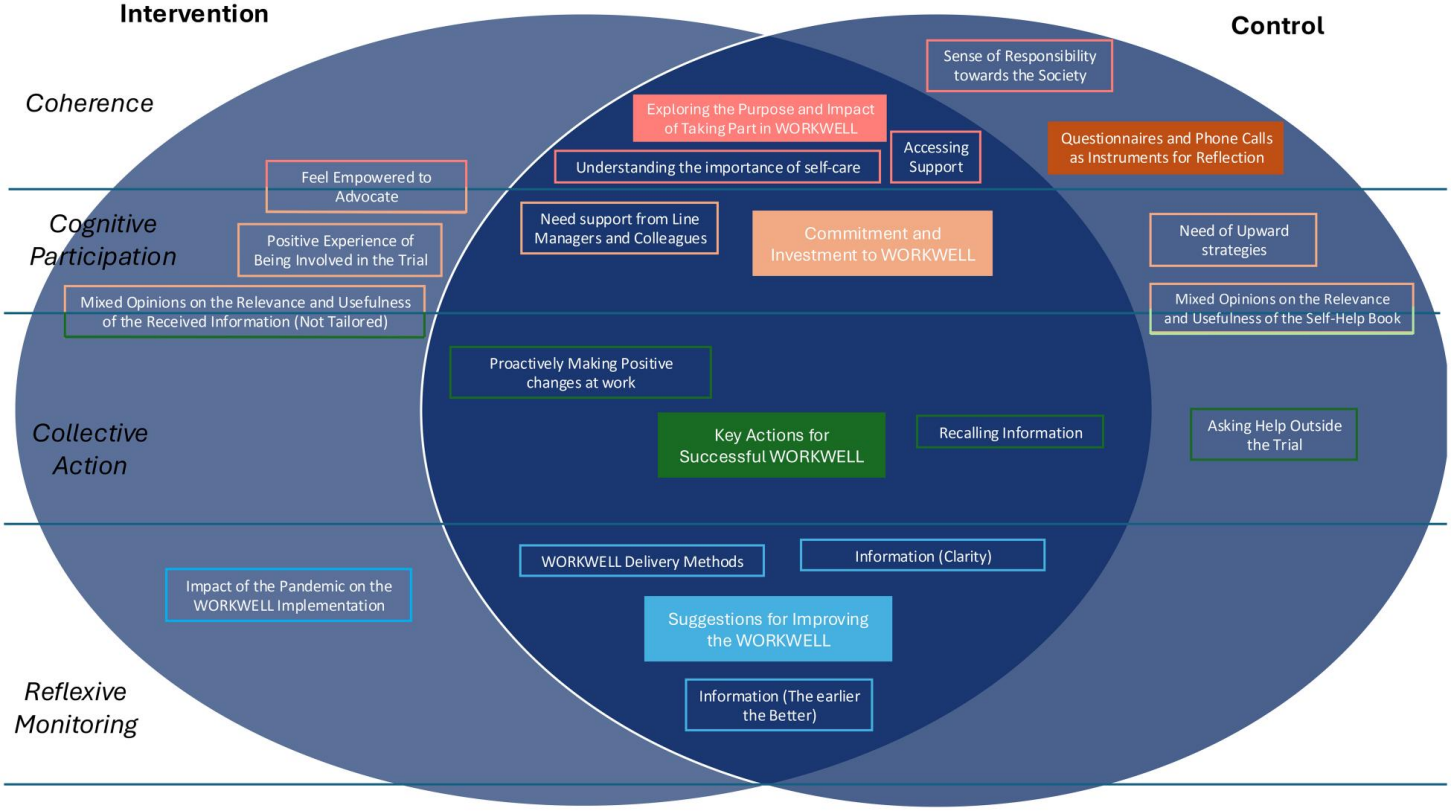
Themes and subthemes following the NPT framework. In the picture, the five main themes are represented in filled coloured boxes. Subthemes are shown in unfilled boxes of the matching-colour themes. NPT: Normalization Process Theory

The COVID-19 pandemic impacted the delivery of the intervention, with only 27% of intervention participants completing treatment before the trial was paused in March 2020. The remaining intervention participants completed (or started and completed) their treatment after the trial was restarted in June 2020, with significant adaptations made to the intervention [14]. These adaptations included a shift to remote delivery, allowing participants to engage with OTs through virtual consultations instead of in-person sessions. Additionally, electronic data capture replaced paper-based assessments, streamlining data collection and improving efficiency. New recruitment and consent procedures were introduced to address challenges posed by NHS site closures and staff redeployment, ensuring continued participant enrolment. These modifications enabled the trial to overcome logistical barriers while maintaining intervention integrity and accessibility [14]. Five secondary themes were created by clustering the primary themes and subthemes (Supplementary Tables S2–S4, available at *Rheumatology Advances in Practice* online) under the NPT framework (Fig. 1).

These themes were common among the different groups at the different follow-ups but with nuances between the intervention and the control groups, as highlighted by the subthemes. Table 4 reports the themes and subthemes with illustrative quotes.

**Table 4.** Secondary themes following the NPT constructs

NPT constructs	Themes	Subthemes	Illustrative quotes
Coherence	Theme 1: ‘Exploring the Purpose and Impact of Taking Part in WORKWELL’	Understanding the Importance of Self-Care (Both Groups)	Joanie (Control, 36 months)—*It was definitely worthwhile doing and it did make me realise how to look after myself.* Harvey (Intervention, 12 months)—*[…] so it was all about challenging my norm, which is what she [The OT] did.*
		Accessing Support (Both Groups)	Janice (Control, 12 months)—*And especially during the pandemic when it was so hard to get in touch with doctors or nurses or get advice, you know, it was helpful.* V18 (Intervention, 12 months, PPI)—*the support and the understanding that I’ve received have been exceptional […].*
		Feel Empowered to Advocate (Intervention Group)	Leanne (Intervention, 36 months)—*It really did help me because I didn't realise just how much I was entitled.*
		Sense of Responsibility towards Society (Control Group)	Liz (Control, 12 months)—*Well, to see if it—the trial can help other people who have arthritis […].*
	Theme 2: ‘Questionnaires and Phone Calls as Instruments for Reflection’		V02 (Control, 12 months, PPI)—*It allowed me, in a selfish way, to reflect on actually how I was feeling […].* Karen (Control, 36 months)—*[…] You’re just kind of paying attention to what’s happening?* V11 (Control, 12 months, PPI)—*Increased my awareness … more confident…. It was nice to get phone calls and have…. Human contact.* V11
Cognitive participation	Theme 3: ‘Commitment and Investment to WORKWELL’	Need Support from line Managers and Colleagues (Both Groups)	Mary (Intervention, 12 months)—*[…] I’m not sure, even after this report that’s sent to them [Line managers], how much will be done. It might be done initially, but it won’t be then checked up or continued.* Mavis (Control, 12 months)—*But on the surface, there’s always we—you know, support and, and putting in, erm, you know, adjustments, reasonable adjustments and things like that, but the undercurrent is very different.*
		Mixed Opinions on the Relevance and Usefulness of the Received Information (Intervention Group)	Mary (Intervention, 12 months)—*We did talk about that. Which, you know, is great in theory, but in practice…* Hayley (Intervention, 36 months)—*So a lot of the things that were suggested to me were things that I was doing almost naturally.* Phoebe (Intervention, 36 months)—*I didn’t really receive an awful lot of advice.*
		Positive Experience of Being Involved in the Trial (Intervention Group)	Kacey (Intervention, 12 months)—*I feel like, if I was just left to my own devices, I wouldn’t be able to find, I don’t think, the suitable advice that’s out there for me.* Pam (Intervention, 36 months)—*Absolutely. Completely from the handbook and the information that I received to, the one-on-one sessions I had with the OT, absolutely, and, have continued using that, up to this day.*
		Mixed Opinions on the Relevance and Usefulness of the Self-Help Book (Control Group)	Dani (Control, 12 months)—*[…] I have had rheumatoid arthritis for many, many years […]. I’ve heard all this before, and it’s common sense, really.* Diane (Control, 36 months)—*Yeah, so for me, it just gave me a huge amount of awareness […]*
		Need of Upward Strategies (Control Group)	Karen (Control, 36 months)—*It would be good if there was more help, not, not from, like, you guys but, like, government help from a, uh, knowing where to go kind of situation*
Collective action	Theme 4: ‘Key Actions for Successful WORKWELL’	Proactively Making Positive Changes at Work (Both Groups)	Sally (Intervention, 12 months)—*[…] I can go home a little bit earlier and I’ve kind of got that in my head now that yes, that’s acceptable. Whereas before […] I would never have thought about doing that.* Joy (Control, 36 months)—*It’s trying to help yourself, yes, and listen to my body I guess, instead of ignoring it.*
		Recalling Information (Both Groups)	Rose (Intervention, 36 months)—*[…] but as the time’s gone on it starts to wane a bit […]* Grace (Control, 36 months)—*I don’t think I’ve got that self-help*… *Have I? If I have, I haven’t read it. Sorry.*
		Asking Help Outside the Trial (Control Group)	Joy (Control, 36 months)—*On top of the pack, I had a lot of counselling, as well.* Brenda (Control, 36 months)—*But, I had a fantastic consultant in the early days who had a really positive mindset.*
Reflexive monitoring	Theme 5: ‘Suggestions for Improving WORKWELL’	WORKWELL Delivery Methods (Both Groups)	Liz (Control, 12 months)—*Because I would then save it and go back to it. Whereas with the paper I tend to put it away.* Patricia (Intervention, 12 months)—*I think perhaps if, with the occupational therapist, if I could have done like a video link.*
		Information—the Earlier, the Better (Both Groups)	Norma (Intervention, 12 months)—*The earlier, the better* Mavis (Control, 12 months)—*Well, the sooner the better, really […]*
		Information (Clarity) (Both Groups)	Harvey (Intervention, 12 months)—[…] *the questionnaire, the follow-up questionnaires, they are a bit painful.* Niamh (Control, 12 months)—*I did read it when I first got it, and to be honest, there wasn’t a lot of information in there that was new to me.*
		Impact of the Pandemic on the WORKWELL Implementation (Intervention)	Patricia (Intervention, 12 months)—*So me job, it’s kind of evolved into all sorts of different things now, from what it used to be and what my job was prior to, you know, when we had the first lockdown from the pandemic.*

NPT: Normalization Process Theory; PPI: patient and public involvement.

### Coherence

Under this NPT construct, we clustered primary themes and subthemes that explained how participants made sense of and derived meaning from the WORKWELL Trial into two secondary themes.

#### Theme 1: ‘Exploring the Purpose and Impact of Taking Part in WORKWELL’

Both the intervention and control groups viewed the trial as an opportunity to understand the importance of self-care (subtheme: ‘Understanding the Importance of Self-Care’), and accepting their diagnosis that was seen as a first step to engaging with the trial, which was also expressed in the theme ‘Commitment and Investment to WORKWELL’ (NPT Construct: Cognitive Participation).

Additionally, they valued the trial for providing access to support (subtheme: ‘Accessing Support’), a critical aspect of their experience. For the intervention group, this understanding was coupled with a sense of empowerment to advocate for workplace accommodations (subtheme: ‘Feel Empowered to Advocate’). This sentiment increased participants’ willingness to engage with the trial, bridging the first and third themes, ‘Commitment and Investment to WORKWELL’. Meanwhile, participants in the control group expressed a ‘Sense of Responsibility towards Society’, seeing their participation as contributing to research that could benefit others with similar issues.

#### Theme 2: ‘Questionnaires and Phone Calls as Instruments for Reflection’

In this secondary theme, the control group highlighted the importance of completing questionnaires and receiving phone calls and emails during the trial. They viewed these activities as tools for self-reflection and treatment. They explained that taking a moment to reflect on their condition, as they are generally ‘wrapped up in everyday life’, made them feel more informed.

### Cognitive participation

Under this NPT construct, we clustered the primary themes that explained how participants committed to and engaged with the intervention into one secondary theme.

#### Theme 3: ‘Commitment and Investment to WORKWELL’

Both groups expressed the need for support from their line managers and colleagues to commit and engage fully with the trial (subtheme: ‘Need Support from Line Managers and Colleagues’). In general, participants highlighted that their motivation to engage with the intervention depended on the relevance and usefulness of the information and materials provided, having an impact on the participant’s ability to make the intervention work, therefore overlapping with the theme: ‘Key Actions for Successful WORKWELL (NPT Construct: Collective Action)’. Specifically, the intervention group had mixed opinions about the relevance and usefulness of the advice given by the OTs (subtheme: ‘Mixed Opinions on the Relevance and Usefulness of the Received Information’) (not tailored) as they perceived that some recommendations were too broad or already known. Despite these mixed opinions, the intervention group generally reported a positive experience of involvement in the trial (subtheme: ‘Positive Experience of Being Involved in the Trial’).

Accordingly, the control group shared mixed opinions about the information in the self-help book (subtheme: ‘Mixed Opinions on the Relevance and Usefulness of the Self-Help Book’). Additionally, a participant mentioned a need for more upward support, stating, ‘It would be good if there was more help, not, not from, like, you guys but, like, government help from a, uh, knowing where to go kind of situation’ (subtheme: ‘Need of Upward Strategies’).

### Collective action

Under this NPT construct, we clustered primary themes revolving around participants’ discussions about the actions necessary to make the intervention effective into one secondary theme.

#### Theme 4: ‘Key Actions for Successful WORKWELL’

Both groups emphasized the importance of a proactive approach for successfully applying the WORKWELL intervention’s strategies (subtheme: ‘Proactively Making Positive Changes at Work’). However, both groups faced challenges recalling the information provided by the OTs or the self-help book (subtheme: ‘Recalling Information’) at 36 months. The control group also highlighted the importance of seeking additional support outside the trial. They found it fundamental to reach out to external resources such as counsellors, general practitioners (GPs), and OTs not associated with the trial (subtheme: ‘Asking Help Outside the Trial’).

### Reflexive monitoring

Under this NPT construct, we clustered primary themes where participants reflected on their trial experiences and suggested improvements into one secondary theme.

#### Theme 5: ‘Suggestions for Improving WORKWELL’

Both groups stressed the importance of offering flexible delivery methods for WORKWELL interventions, allowing participants to choose between in-person and online options based on their preferences. They suggested introducing a digital version of this programme (subtheme: ‘WORKWELL Delivery Methods’). Additionally, both groups agreed on the significance of timely information delivery, especially for those recently diagnosed (subtheme: ‘Information—The Earlier, the Better’). Both groups also agreed that the provided information was generally clear and in lay terms. However, they found some of the provided information and the questionnaires lengthy and repetitive [subtheme: ‘Information (Clarity)]’. Participants in the intervention group also discussed the challenges posed by the pandemic, such as job changes, increased childcare responsibilities and feelings of isolation. They suggested a need for adaptable strategies within WORKWELL to address these evolving realities (subtheme: ‘Impact of the Pandemic on the WORKWELL Implementation’). There were no differences in participants’ experiences who attended the intervention before and after these practical adaptations were made due to the COVID-19 pandemic.

## Discussion

The findings of this qualitative study, nested in the WORKWELL trial, provide insights into the experiences of individuals with IA enrolled in the trial. A recurring theme was the mixed perception of the intervention’s relevance. While many participants appreciated the support from OTs and the information pack, some found the content insufficiently tailored to their needs. Several participants noted that much of the information was either too general or already known to them. To enhance future interventions, programmes should incorporate more personalized elements, such as tailored guidance based on disease severity, job demands and personal circumstances. While this could pose challenges within the NHS due to resource constraints, integrating digital tools for self-assessment and targeted advice could help address this issue [23]. Additionally, both groups expressed difficulty recalling information after 36 months, indicating a potential need for ongoing support beyond the initial intervention.

The degree to which participants could engage with the WORKWELL trial also depended significantly on the support they received from their workplaces. Many participants highlighted the necessity of buy-in from line managers and colleagues, yet they often encountered superficial support that did not translate into meaningful (or no) workplace accommodations. This lack of understanding was partially perceived as due to a lack of knowledge of IA-related symptoms, especially those invisible (e.g. pain and fatigue), as reported in other long-term conditions [24, 25]. Beyond workplace buy-in, other factors also influenced study outcomes, including the severity and fluctuation of participants’ symptoms, the nature of their job roles and the availability of workplace flexibility. These findings align with previous research indicating that JRVR interventions are most effective when workplace culture and policies actively support employees with long-term conditions [24–26]. Notably, we tried to contact some of the participants’ line managers, but they either did not reply or declined.

Interestingly, participants found value in the reflective aspects of the trial, particularly the patient-reported outcome measures (PROMs) and telephone calls, which helped them track their progress and better understand their condition. Several mechanisms support this process [27]. This process of self-reflection through PROM completion and calls empowers patients, becoming an intervention itself [27]. However, the repetitive nature of these elements was occasionally a point of frustration. This finding suggests that while self-monitoring tools are beneficial, their design should balance engagement and burden [28]. Future research should explore ways to optimize the frequency and format of such tools to enhance user experience. Additionally, there is a need to design effective follow-up mechanisms that could reinforce key messages and improve long-term retention of intervention benefits [29].

The COVID-19 pandemic introduced unique obstacles, particularly in adapting to remote work and digital delivery of services. Participants expressed positive and negative views regarding remote working, which affected their health and productivity differently. While some appreciated the flexibility, others felt isolated or burdened by increased childcare responsibilities. Beyond COVID-19, the shift towards remote and hybrid work remains a key consideration for future JRVR interventions. The findings indicate that interventions must be adaptable to evolving work environments, suggesting that future JRVR programmes should incorporate hybrid models to maximize accessibility and effectiveness. The feedback points to the need for flexibility within JRVR programmes to accommodate changes in the work environment and offer varied delivery methods, which led to the creation of a digital version of the WORKWELL programme (https://www.workwelluk.org/) after the completion of the WORKWELL RCT [30]. Providing digital options and hybrid models could address participants’ preferences [31, 32].

Several limitations to this study should be acknowledged. First, as a nested qualitative study within an RCT, the findings are specific to participants in the WORKWELL trial and may not be transferable to other JRVR programmes. However, key themes, such as the importance of tailored support and workplace engagement, are known to be relevant across similar interventions [33]. Future research should explore how these findings apply to other populations, including those in different employment sectors or healthcare systems. Additionally, the influence of the COVID-19 pandemic on the trial posed significant challenges to participants’ engagement and experiences. Another limitation lies in the data collection method. Although interviews provided valuable insights, reliance on self-reported data could introduce recall bias, particularly regarding the 36-month follow-up. However, the aim of the 36-month follow-up was also to understand which information participants retained over time. Most of our participants were white women with RA, limiting the transferability of our results to other populations. Future research should include a more diverse sample, particularly individuals from different ethnic backgrounds, socio-economic groups and occupational settings. Finally, we interviewed different participants at the two follow-ups, reducing the possibility of comparing data at the two time points. The strengths of this study lie in the use of a structured framework, the high number of interviews that create a unique qualitative data set and the deep PPI involvement in each stage of the research. Additionally, this study highlights gaps in existing research on JRVR interventions, particularly regarding the long-term sustainability of workplace support and the role of digital interventions. Future studies should investigate the long-term impact of tailored digital support tools, explore employer perspectives and assess the cost-effectiveness of digital JRVR interventions within healthcare systems like the NHS.

## Conclusion

In conclusion, the WORKWELL qualitative study sheds light on the complexities of implementing JRVR for individuals with IA, emphasizing the need for tailored, flexible and workplace-integrated approaches. The intervention has demonstrated benefits in supporting participants. However, addressing the variability in individual needs and enhancing workplace involvement could have improved the intervention’s impact. Incorporating more tailored feedback loops, greater flexibility in delivery methods, including digital options, more frequent touchpoints with occupational therapists, and structured follow-ups could have further strengthened its impact. These strategies are potential keys to maximizing the effectiveness and long-term sustainability of JRVR programmes like WORKWELL.

## Supplementary Material

rkaf034_Supplementary_Data

## Data Availability

The data underlying this article will be shared on reasonable request to the corresponding author.
