# Supplementary material for: WORKWELL process evaluation: qualitative data analyses of the participant interviews at 12- and 36-month follow-ups
Source: Rheumatol Adv Pract. 2025 Mar 14;9(2):rkaf034. doi: 10.1093/rap/rkaf034 (PMC11930348; doi:10.1093/rap/rkaf034)
Supplement: rkaf034_Supplementary_Data [file rkaf034_supplementary_data.zip › Supplementary Table S1 Interview Guides.docx]

Supplementary Table S1 Interview Guides

| **Interview Phases** | Questions |
| --- | --- |
| *Intervention Group, 12-month Follow-up (Researcher)* | |
| **Starter question** | - Can you tell me what your job is, and can you describe your workday? |
| **Treatment programme and components** | - Thinking back to the first meeting you had with your occupational therapist for the WORKWELL programme, can you walk me through what happened at your first meeting? - Can you describe the work problems you discussed at your first meeting? - Can you describe what happened in the following meetings? - Can you describe the advice or support they gave you for each of your work problems? |
| **Personal Factors** | - Have you made any practical changes in your workplace since your appointments with the occupational therapist?   - If you have made changes, please can you describe the changes you have made at work?   - Can you describe what helped you make these changes? [     - If you have not made changes, please can we explore this further – why do you feel you have not been able to make any practical changes? - Are there any concerns you have about continuing to work in future? If so, can you describe your concerns? |
| **External Factors - Relationships** | - Can you describe what the relationship was like between you and your occupational therapist? - What support have you received from your employer/line manager to help you stay in work? - Can you describe any support you have received from your work colleagues? Did this come about because of having the workwell appointments? - Are there any changes you have made to your home routines to help you stay in work? Did this come about because of having the workwell appointments? - Are there any changes you have made to your social / leisure activities to help you stay in work? Did this come about because of having the workwell appointments? - Are there any other factors which have helped? |
| **Paused mid-treatment*** | - Were your appointments with the occupational therapist affected by the COVID-pandemic / trial being paused for a period?   - If yes, can you describe what aspects of your appointment(s) were affected?   - Were you able to make changes at your workplace before the trial paused? If you were, how useful were these changes?   - Can you describe what the experience was like going back to see the occupational therapist after the paused period? Did you have any new work problems that you needed to discuss? Has any advice changed? |
| **Setting** | - Can you tell me what the experience of getting to your WORKWELL appointments was like? - What are your views about the way the appointments were delivered? i.e. can you describe how your experiences with the face-to-face / telephone / video appointments?   - Would you have preferred another method of delivery with your appointments and why? E.g. if you had face-to-face, would you have preferred telephone? |
| **Suitability of the programme** | - Do you think it was appropriate for the Rheumatology team to provide you with help about work issues? - † When is the appropriate time to introduce Workwell to patients post diagnosis? - Was the work programme you received right for you? Why? Why not? - Was the investment in terms of your time worthwhile? Why? Why not? - Can you describe any advice / assistance you felt were unnecessary, or would have liked to receive but did not |
| **Improvements to the intervention** | - What could be improved or done differently? - Are there any other comments you would like to make about the work support provided? |
| *Control Group, 12-month follow-up (Researcher)* | |
| **Starter question** | - Can you tell me what your job is, and can you describe your workday?   What initially interested you about the study? |
| **Intervention** | - What did you think about the self-help pack you received? These were: 2 Working with Arthritis booklets, I want to work booklet, and a yellow Equality Act booklet.   - Did you read them? If not, may I ask why not?   - † (if they read booklets): what motivated you to read/use the booklets?   - † how did you use these booklets? E.g., highlighting, making notes?   - Do you still have these to refer in the future if the need arises? † Why? - What changes (if any) did you make to help you stay in work during your time in the trial?   - Were the changes made initiated by your involvement in the trial?   - *If not due to trial involvement, what circumstances led to making these changes?*   *Have you discussed any work advice/ changes with your employer/line manager? If so, can you describe what happened?* |
| **Individuals’ preferences** | - † What method of receiving these kinds of self-help information do you prefer: hard copy booklets or online weblinks sent to your email? Why?   † How long after being diagnosis of your condition do you think would be the best time to approach patients with the self-help pack information? |
| **Questions on the Booklet if not read** | - If you have not read the self-help booklets and/or made changes, can we explore why?   - *(Prompts) Have you explored different changes you can make in your work previously for example? Or have you developed your own strategies to cope with work difficulties you are experiencing due to arthritis?*   *† Were these changes in place before you entered the trial or did reading the self-help pack led you to making these changes?* |
| **Effect(s) of the pandemic*** | - During your time in the trial, did the pandemic / lock downs affect your ability to work? (e.g., were you able to work from home, were you still required to go into your workplace or were you furloughed / shielding?)   - *If yes, can you describe what effects the pandemic had on your ability to work and your arthritis? e.g., if worked from home, how did you find the impact of this on your arthritis?*   *Were you able to make changes at your workplace during the pandemic? If you were, how useful were these changes? If not, why not?* |
| **Ending questions** | - Are there any concerns you have about continuing to work in future? If so, can you describe your concerns?   Are there any other comments you would like to make about taking part in the trial? |
| *Control and Intervention Group, 12-month follow-up (PPI)* | |
|  | 1. Why did you decide to agree to take part in the Workwell study? 2. How clear was the information contained in the Workwell study documents and paperwork? 3. What went well for you in the Workwell study? 4. What could have been done better? 5. How did you find the overall delivery of the study? 6. What was your experience of taking part in the Workwell Study? 7. If the participant was in the Treatment Group: Have you any thoughts as to how the Workwell treatment could be implemented within the NHS, if the study is successful? 8. Is there anything else you feel it would be relevant to mention about the study? |
| *Control and Intervention Group, 36-month follow-up (Researcher)* | |
| **Starter question** | - Could you tell me a little bit about your current work situation? What do you do for a living? - The advice you have received in the trial made any difference to how you are coping at work now?’   *‘Is your work status changed since the trial, and if so, in which way?’* |
| **If the participants is still in work:** | - Do you remember the advice you were given and what changes you have made because of this? - Have you made any additional changes in your workplace/ to your work practice since you left the trial? - Did you feel you had the ability, resources, and confidence to take forward the advice given in this trial? Any strategies that you use whilst working? - Something about work/life balance here? - How confident do you feel in continuing to work in the future?   Are there any other comments you would like to make about the work support provided?’ |
| **If the participants is no longer in work/unemployed/retired early:** | - In your view, what were the reasons for you stopping work? - What factors led to you stopping work? - Further prompt questions will be used, as necessary, to explore specific aspects to ensure similar types of information are collected from all participants, (with additional suggestions of examples given for each if the participant needs further prompts): - Did any practical factors contribute to you leaving work? - Did any changes in managing your home routines or social / leisure activities contribute to your leaving? - Did difficulties with managing your health and arthritis symptoms contribute? - work/life balance? - Were you able to discuss your condition with your employer? Were you able to discuss any possible work advice/ changes with them? – How’s your relationship with your employer? - Were you able to make any practical changes in your workplace since your participation in the trial before you stopped working? - Do you feel anything could have been done differently or improved, in terms of the WORKWELL programme, that you think may have helped you stay working? - Was there any advice/ assistance you would have liked to receive but did not?   Was there any advice/ assistance that you consider were unnecessary? If yes, what and why?   - Do you plan to return to employment in future?   Are there any other comments you would like to make about the work support provided in this trial? |

Legend: •, questions asked to everyone; o, follow-up questions; * additional questions after the COVID-19 pandemic outbreak, may also apply to participants who were furloughed/shielded/income support; † questions not presented in the original interview guide but added afterwards (Sep 2022);
