# Supplementary material for: WORKWELL process evaluation: qualitative data analyses of the participant interviews at 12- and 36-month follow-ups
Source: Rheumatol Adv Pract. 2025 Mar 14;9(2):rkaf034. doi: 10.1093/rap/rkaf034 (PMC11930348; doi:10.1093/rap/rkaf034)
Supplement: rkaf034_Supplementary_Data [file rkaf034_supplementary_data.zip › Supplementary Table S2 Primary Themes Intervention and Control Group 12-month Follow-Up (Researcher).docx]

Supplementary Table S2 Primary Themes 12-month Follow-Up (Researchers)

| **Theme** | **Sub-themes** | **Quotes** |
| --- | --- | --- |
| ***Intervention Group*** | | |
| **Perception of Workwell** | **Acceptance of condition and need for change** | **Sally** – “If I'd have gone and not been prepared to try the things that she was suggesting, there was absolutely no point my going in the first place.”  **Tom** – “I got quite a bit out of it. And it, it, with my works as well, it highlighted that it wasn't just something I was going to sit back and let carry on. It was showing that I was proactive by getting some, some additional help, to help me stay in work.”  **Jodie –** “We’ve talked about basically acceptance that it … it’s not that it’s bad, it’s just it’s changed. It’s just different, and it’s just a different way of working, or a different way of life, and you can still have a full life, but you know, you … you just have to pace yourself a little bit. And … and the main thing is, um, which he keeps saying is, it’s recognising that if you’re having a bad day and you need a duvet day, have a duvet day.” |
|  | **Values gained from the Workwell** | **Harvey** – “You know, again, not, not taking regular breaks. You know, not eating regularly and things like that. Which, which I know it sounds ridiculous, because it’s all the right things to do. But doing it for years that becomes my norm, so it was all about challenging my norm, which is what she did, to be honest with you.”  **Maisie** – “It's opened my eyes as well, just … in the beginning I think you don't realise how much you should be doing and how much help is out there and how much help you can ask for… I wouldn't have applied for Access to Work had I not had that conversation with her [the occupational therapist]”  **Kacey** – “I feel like, if I was just left to my own devices, I wouldn’t be able to find, I don’t think, the suitable advice that’s out there for me. Um, having that expertise, um, just opened my eyes a bit to, like, what is out there and what is available. So, I definitely do think, yeah, having someone like the OT to go and, you know, chat to about arthritis things and working is very, very, um, very useful.”  **Max** – “I was just pleased someone had listened to me and I got some answers, that was what I was pleased about.”  **Jodie** – “I remember the first session with the OT. Um, I was … err, she chose … gave me a lifeline really. Um, I think it’s the best way I can describe it, because my world had just turned upside down, and … and *the* world had turned upside down, um, and she was…and she gave me hope that we could work through it and, um, there is light at the end of the tunnel. She gave me real hope at that first session.”  **Laura** – “So we had all different conversations about, you know, like different tactics to get out of, um, out of work on time really. And it was very help- … you know, it did make me think about it. You know, it was good to talk over it with somebody else, you know, and, and to raise, you know, um, possibilities of doing, you know, can you do this, can you do that?”  **Kacey** – “I think having the OT there just … again, just gave me options, er, maybe things that I hadn’t thought about, or gave me options to explore a bit more…It was just good to have someone there to just be, like, ‘well here are your options, have you thought about this?’ Um, generally just having that two-way conversation.” |
| **Commitment and investment to Workwell** | **Proactively and thoughtfully making changes and requests for work adaptations** | **Sally** – “I kind of now, where I would never have done it before, I would always … if I wasn't very busy, I wouldn't leave early, I wouldn't do things just in case somebody called or somebody came in. Erm, where now, if I know I've got a real quiet afternoon… well, I'm going to go home. You know, I'm not going to knock myself out just to sit there to do nothing. I can go home a little bit earlier and I've kind of got that in my head now that yes, that's acceptable. Whereas before, it was never … I would never have thought about doing that. You know, so I think the way I'm thinking about work has changed.”  **Laura** – “It also has helped, like the thought of it, the benefits and the risks. Um, where, I don't kind of, you know, put myself forward for so much anymore, I just think, sometimes you're not really thanked for it. You know. [laughs] Um, you know, what will it … you know, I could do that and I could do that extra work, but, you know, it would be easier not to really. I don't know whether that sounds a bit lazy. But it … it kind of made me think of that, yeah, why should I… I'm a bit too enthusiastic really. [laughs] So to, you know, calm down and pull back a bit. So, it did make me … although those weren't things she directly said, it did make me think, if that makes sense.”  **Trinny** – “Actually I’ve actually slowed down a lot. Yeah. Not working at such a fast tempo. And I think … I think that that’s helped as well. Um, I think the … just taking time out to plan what I’m doing better. Whereas before, it was just like, um, habit. I wasn’t actually thinking about the effect that that was having on my body. If you see what I mean. But if you just step back and think, you know, ‘Oh, maybe I could do this a different way, or that a different way, or maybe I should do that, you know? Yeah, that sort of thing…it’s made me think more about my illness and less about my work…if you see what I mean.”  **Eddy** – “Um, there was, I think I, while still feeling tired at the end of a working day, I probably felt a better sense of achievement in the sense that I would look and see that I had actually completed quite a number of tasks and it wasn’t, um, such a mountain to climb. Um, we do look to see what other ways we can do things and whether or not we can use different tools to make it easier.” |
|  | **Tailoring the intervention** | **Eddy** – “There were some things that she suggested that tried and I tried and they didn’t work and it was like well, that’s okay, didn’t work for you, but it might work for somebody else, we can try other things, although for the life of me I can’t remember what it was. But as regards, you know, was there something else I could have done with, to be honest with you I don’t know what I don’t know. And therefore I don’t know what else was available or what else I could have considered.”  **Mary** – “We did talk about that. Which, you know, is great in theory, but in practice, when you are teaching a class, when do you get a break? And we talked about, you know, things like mindfulness, but I suffer from chronic fatigue and I go to the chronic fatigue clinic, I’ve been seeing them for over a year and they go through these sorts of things to try and help, but in practice, in your day to day work, there isn’t, there isn’t time or space for it.”  **Kacey** – “Um, so, the things that we discussed were maybe trying some meditation, um, which I … I struggle with. I … I kind of don’t get it or I just … I can’t seem to, like, switch off, so meditation is, er, quite difficult. I have tried it, but I find it quite difficult to keep at it, so that one’s been parked for the moment.” |
| **Practicalities and implementation of Workwell** | **Workwell** **delivery methods** | **Harvey** – “I think, I think they were certainly long enough. Um, and we were sort of doing it once a month. Uh, which, which, which I think, sort of looking back on the sort of targets that I was set… I think was probably about the right length of time, to be honest with you. Anything less, I probably wouldn't have had sufficient time to really sort of … sort of get, sort of get heavily involved in sort of changing my habits and changing my ways, etc. Uh, so yeah, I think they were fine, and I think timing, she was really good. Um, she would always sort of make sure the timing suited me because I have, yeah, because I have particular days in the week, I have particular parts of the day during the week where, um, yeah, I'm able to fit things in.”  **Sally** – “Nobody has told me about any sort of aids that I could get, any help that I could get… with my work. Erm, even rheumatology haven't mentioned anything in the past. You know, they know what I do for a living, but I think they're more sort of … yes, your medical side, as in dealing with the immediate issues… rather than the wider effects of your issues. So, in order for rheumatology to be able to work alongside the occupational therapy… I think was really good.”  **Maisie** – “Erm, I think without that [rheumatology team], erm, I wouldn't have known what options were out there for me to help me with work. Err, and I'd have been … things would've been a lot more difficult for me… had that not happened. And they prompted me to go for Access to Work, which again I would never have done. And it's to point out some of your rights, as well. To say, look actually, you have got a long-term condition. Err, yes you can get help.”  **Mary** – “Probably, I think better from the workplace. And it would have been good to have maybe that meeting, that visit, at the beginning, and then my work and negotiate the meetings in work, and then, you know, things could have, I felt like things could have been then happening, things could have been intervened, things could have been fixed and then see how things are. And do, you know, just have that regular check in with things, with my work manager.”  **Katherine** – “Because … because you feel like the person … because, because I've got the rheumatoid arthritis…and it was being held in the … in the rheumatology department. You then think … then you've a sense, sense of like, well, the person is in rheumatology, so they know all about it. Or they know roughly the condition I'm going through. If I was sent to … if I was sent to somewhere else completely. Completely different, like … like dialysis, somewhere over there…I'd feel like, do you know anything about rheumatology?”  **Molly –** “When you go to your like rheumatology appointments they don’t have time to go into depths and things like that with you. Um, I, I sort of , I enjoyed that part with [the Workwell occupational therapist] more than I enjoy it with my consultant. I don’t, sort of, have the same…I don’t, I don’t, I, I didn’t feel as much support from him as I do with, with these. Because they, when you go for your consultant appointments they just, um, do the normal chats like they do every, every time you go ask the questions. Then they’re rushing to get you in and out basically. Whereas when you’ve got these appointments they actually take their time to ask you questions and listen to what you’re saying. And then providing you with a, um, what’s the word. If you’ve got a particular problem…they’d provide a, um, some helpful hints and tips and things like that. They understand that.”  **Katherine –** “The consultant will go, yes, looked at this, looked at that, fine, put her on these meds, she’ll be fine, see her in six months. They, they can be very much like that. Sometimes … they don't go, you know, are you finding it difficult working? Are you having problems when you work with your bones or anything like that? Or are you taking sick because you can't get out of bed or something like that? They don't … they never ask that. So maybe the … maybe the consultants or something might want to ask that.”  **Trinny –** “So, once that person’s diagnosed then… the Rheumatoid Department triggers something and lets you know that there’s somebody that’s full-time working, you know. And then you can work with them…And that there’s somebody there to help them, support them within that.” |
|  | **Relationships** **with line manager: Mixed confidence to request changes** | **Harvey** – “Um, the, um, the Dragon software and the trackable mouse, I just got through work. I just explained to my boss that, yeah, that, you know, that I'd been, you know, advised by the therapist to get it and they're fine, yeah, he just said, yeah, get it.”  **Laura** – “Yes. Yes. Yeah. So we had several, um, goals for kind of cutting down the work. Um, there was a suggestion you could work from home once, um, you know, an afternoon a week, which I did take to my line manager.”  **Mary** – “But now, I know that the OT sent a report that I have given, because you know with her suggestions, and given that to my boss and my direct manager and they are in meetings about making something happen, so that’s good.”  **Mary** – “If we’re trying to increase awareness of what people with rheumatoid arthritis or with arthritis need in the workplace, it’s just a shame that nothing is, nothing’s changed, nothing’s done and I’m not sure, even after this report that’s sent to them, how much will be done. It might be redone initially, but it won’t be then checked up or continued.”  **Eddy** – “Um, and um I mean I’ve got a very empathetic boss who, um, I have to be careful if I say oh, I could do with something like this and before I know it, he’s gone out and bought something, but it’s like the most expensive thing ever and so you’ve got to be like really carefully you don’t say – because I said I could do with a table that lifted up and down, I could adjust it and he went out and spent nearly five grand on this massive thing, a table half the size would have been what I was after, but no, he had to go and get the biggest, the best, it was like there you go, that’ll make your life easier, I am like yeah, thanks for that. And everyone’s looking at me and I am thinking I really didn’t need this, you know. And they still rib me about it now, you know.” |
| **Evaluation of Workwell** | **Worthwhile** | **Harvey –** “Yeah. Yeah, it was [worth the time investment]. Yeah, it was. Yeah. Yeah, certainly attending the sessions with the therapist. Uh, because, I'll be quite honest, if I didn't think it was worth the effort of having to get to the hospital, the effort of parking and getting in, etc., I'd have pulled the plug on it, I wouldn't have done it, to be honest with you.”  **Laura** – “But it was, but it was worth it really, you know, I kept going. And it was … I work on one hospital site and she was on another hospital site on the other side of the, the town, um, which was a bit unfortunate, but… You know, it was worth going really.”  **Kacey -** Yeah, definitely. Um, I feel like, if I was just left to my own devices, I wouldn’t be able to find, I don’t think, the suitable advice that’s out there for me. Um, having that expertise, um, just opened my eyes a bit to, like, what is out there and what is available. So, I definitely do think, yeah, having someone like the OT to go and, you know, chat to about arthritis things and working is very, very, um, very useful. |
|  | **When to Introduce Workwell to individuals** | **Laura –** “Um, yes, I think so. Um, yeah. Um, the only thing I can think of, I did feel a little bit guilty, because I was getting better as time went on. Um, if I'd had it [Workwell programme] a bit earlier, um, you know, after my diagnosis, that would probably have been more beneficial for me. I felt a bit bad that I was already kind of, I don't know, on the way to getting back to, you know, kind of normal. Whereas if you had only been newly diagnosed or you'd only had it for kind of six months, and you were still struggling working, with work, it would have been more helpful, you’d have got more out of it. So it was a bit late in the day for me. But it was, uh, still very helpful. ” **Jodie –** “I … I think it should … well, maybe not necessarily straight away, because I think there should be a short period of … because you’re going for bloods and you’re trying to sort your drugs out, and it’s all like, ‘Oh my word’. So, I think if it’s very early on you’re not really going to take it in so much. You’re not at that point where you’re ready to listen and get the benefit of it. But I would say definitely within a couple of months of diagnosis.”  **Norma** – “The earlier the better” |
|  | **Suggestions for improvement and concerns** | **Harvey – “**Um, not, yeah, honestly, yeah, I guess, other than, other than perhaps being more local to where, to where I am. Because again, thinking about people with arthritis, you know, it is, it is tricky to drive and I can't … or unfortunately, you know, I could never rely on my wife to take me, because she was always working herself. So, so maybe if it had been a little bit more local, it would have been, yeah, an easier undertaking to get there. But, you know, it is what it is, I realise. And, and as it transpired, the actual therapist lived very close to me.”  **Laura** – “No. Um, I don't know. Maybe, I mean I'm not saying that … yeah, maybe like a phone conversation between appointments, you know, like update or something like that. Um, just to kind of keep you on track really.”  **Mary** – “I don’t know, it would have been nice to try and keep, you know, like some people keep a diary or just contribute on a webinar or something like that every so often, so you would meet other people as well. I haven’t spoken to any other person who’s done this trial, so I don’t know how it’s been beneficial to them, I don’t know if they had meetings in the hospital, I don’t know, you know, there’s no, it’s just me alone isn’t it.”  **Patricia** – “Um, I think perhaps if, with the occupational therapist, if I could have done like a video link.”  **Harvey – “**All I would … yeah, all I would say, the questionnaire, the follow-up questionnaires, they are a bit painful.”  **Maisie – “**Yes, I do, yeah, yeah. I have to say the initial [laughs] surveys were taking … they took me a lot, lot, lot longer than was expected. [Laughs]”  **Eddy** – “I don’t think Work Well is going to work for everyone and I don’t think every business is going to sort of want to adopt it, because they will see it as a cost on their business.” |
| **Impact of the COVID-19 pandemic on the trial** | **Positive and negative effects of pandemic on Workwell implementation** | **Marie – “**Yeah, yeah, because I suppose, with Covid, it’s … um, we all had to adapt in some way, didn’t we?”  **Patricia – “**So me job, it’s kind of evolved into all sorts of different things now, from what it used to be and what my job was prior to, you know, when we had the first lockdown from the pandemic.”  **Mary – “**You know, I think the isolation of not being in an office and you know just being round a six year old and an eight year old has taken its toll really. And also trying to keep them occupied, but being vulnerable, so, you know, not going out and not, and obviously I’m separated from their dad, and so, you know, he wants, it’s, you know, he’s been away and then wants to see them and it’s that whole parent thing and trying to sort out childcare and taking them to school with masks on and, yes, everybody’s been through this, it’s not just me, but it’s really been difficult.”  **Maisie** – “Yes, yeah, yeah, I have, yeah, yeah. Yes, definitely. The getting up regularly, erm, is something, erm, so yes, I do make sure I'm up and down. It's a bit more challenging with COVID because you're limited to how many people can go in the staffroom at a time, that sort of thing. Erm, you're limited to where you can walk, [laughs] you know, being careful how many people I'm coming into contact with.”  **Katherine –** “with being able to work at home for two days a week, I could take more breaks. Because even if I just sat at my desk and didn't do any … sit at my desk and don't do anything. Apart from something like drink a cup of tea or something like that. At least I was getting a break.”  **Katherine –** “they've not made it compulsory. But I thought I better … I better start going back in. So because … yeah … so, I found that a bit stressful, I must admit. Because I'm so used to working at home. I've been at home for like two years. So I've been able to take the breaks I have and I've not had actually … I've not had many flare-ups at all. Because … because I've been able to sort of work at my own pace. I still get the work done. But I can work at my own pace, and I haven't got to, um, carry everything to the train station, I haven't got to keep, you know, going up and down the stairs and things like that. So … but it was … and I've got my own sort of set up and, and things like that. So I'm sort of stress free with it. So I've not actually found it very stressful at all, I've got quite used to it. To be honest. So I went back, back in the office and then got stressed.”  **Jodie –** “There’s the flexibility, definitely, um, to the time I start in the morning. Because mornings are usually the hardest, to get going in the morning. Um, and a bit of brain fog, things like that. So, there’s that, but also there’s you don’t have to put on a show, because nobody can see you. So, if you’re not feeling very well, it’s not as if you’re sat in an office and you don’t want to look like you’re miserable or in pain or … [laughs] Or, you know, you … it … it just … something silly gets on top of you and you just want to go and have a cry. You can have a cry and come back to your computer and…you’re fine. There’s not that added peer pressure of being around people.”  **Mary** – “I know that I’ve struggled a lot work, a lot more from working from home, definitely. That’s been a real struggle and everybody’s sort of just had to get on with it. And it’s been for a long time, if you think about it…It’s been since last March…I think I just experienced a lot more flares recently, a lot more fatigue, to the point like, you know, where I am sleeping for 15 to 16 hours at a time and, yes… Yes. And I think trying to get hold of doctors, consultants, when you are having a flare has been a real nightmare too. And then it takes days before somebody can respond and then get a message to somebody else and then try and, you know, just trying to go and have steroid injection’s just been very difficult.”  **Jodie –** “I was saying to them it’s a bit like a double-edged sword. So, I think, because I was diagnosed in the February of lockdown happening in March, so … and it all happened within four weeks, I had no symptoms to then all the symptoms… I think if I’d been going into work, I think I would have missed a lot of work, because I … I wouldn’t have been able to work as much. But on the other side, I’ve worked more than I think I should have. And I was saying, on days where you would normally think about getting in the car and driving to work, I’m not well enough to do that, I’ll have the day off. But because I’m just coming downstairs and sitting in front of a computer, you think oh it'll be alright, I’ll just work. Yeah, it’s … I don’t know how to … it’s great, but it’s not great [laughs].” |
|  | **Timing of the trial** | **Harvey** – “So no, not really. You know, yeah, so there's nothing that really stands out. You know, I … whether I'd be as positive about it if, if we were trying to do this during lockdown and it was phone calls and things like that, I don't think it would have been as successful for me and I don't think it would have been as, as enjoyable for me either.  **Mary** – “Yes. At the last, you know, the phone interview we had, I wasn’t in a good state and…Um, you know, she was like well maybe look for a different type of job, but that’s all very good and well, but so many people are unemployed right now and it’s not easy to find work which can accommodate my hours and my needs as a single parent. So you know, again, I understand why she did that. But I also think she’s in a difficult situation, you know.” |
| ***Control Group*** | | |
| **Benefits of Being Involved in the Trial** | **Altruistic (rewarding, satisfaction, sense of responsibility)** | **Janet** – “Because I think anything that can help research, help other people. I don't - I, I've been involved in lots of trials and things at the hospital. They'll ask me if, you know, not - I was gonna say do experiments on me [laughs]! It's not quite like that, but I've done it with when I've had scans and stuff like that when other students are learning. Or I've been used as case studies before to take to conferences. Anything, I'm, I'm always happy to help because it, you know, how do you learn and get to know about things if you don't do research, and then you need people to help in that research?”  **Janice** – “I'd just like to try and help as much as possible to see if there's any way forward with cures or being able to help other people or that, you know? 'Cause it's, it's not a very nice thing to have, obviously.”  **Liz** – “Well, to see if it - the trial can help other people who have arthritis, as well as myself, and the way forward.”  **Mavis** – “For me, I am, erm, very, very passionate about, erm, diversity and keeping people in work. Work is crucial for any health problem, I feel… whether it's physical or mental. Some sort of work gives you so much more than pay… Erm, it's the benefits around, er, feeling useful, needed, er, skills, erm, just, just a feeling of, of being worthwhile.”  **Tina** – “It's about I think going forward and taking part in something that's gonna help other people because I think what you've done over the past 12/18 months is gonna benefit others because it's all… It's about the knowledge and about what works, what doesn't work, and that's really important about taking what works forward.”  **Willow** – “And I did find that [the programme] very helpful, so if I can help anyone in the future how to manage their condition, then I'm all for it.” |
|  | **Accessing Support** | **Janice** – “And espec-, and especially during the pandemic when it was so hard to get in touch with doctors or nurses or get advice, you know, it was, it was helpful.”  **Tina** – “I, I just wanna say I think it - for me it's been really beneficial. I'm really glad I was involved in it. Everyone has been really good from your end with the e-, going from the emails and the phone calls. So everybody has been really supportive all the way through from start to finish.” |
| **Effective Implementation of the Programme** | **Available online (Having the option to have the programme online)** | **Liz** – “Because I would then save it and go back to it. Whereas with, with the paper I tend to put it away. I know where it is, but it, it's the coming back to it.”  **Mavis** – “I, I think that for me personally… a generational thing, but I like hardcopies. Erm, I think both, actually, but bearing in mind that arthritis tends to affect generally older people who wouldn't necessarily, erm, you know, de-, be able to deal so well with online information. Erm… I like a hardcopy in front of me that I can refer to as and when I want to. But…it is a generation - if you had a 20 or 30-year-old in that position they would say the opposite, I'm sure.”  **Niamh** – “I thought the hard copy version was better because, again, being online all the time… Just does my head in. Um, I know it, sort of, saves in terms of printing and paper and postage, and all that sort of thing, but I think, when stuff gets - we, we get so much stuff sent through on email attachments or whatnot that I think you just don't read it…”  **Willow** – “Me, me, me personally, I like a hard copy in front of me. I'm, I would prefer reading in a book than looking online, but having said that, I will do online, but I like the physical copy of something in front of me to actually read, rather than, again, holding my phone is awkward, you know. That's, that's, that's another thing I struggle with, so if I've got my phone or my tablet, which is a bit bigger, but even just holding a phone or a tablet for any length of time I struggle with. So me personally, I like a hard copy. But I should imagine the way the, you know, everyone nowadays, we probably would prefer things online.” |
|  | **Information Timing (Earlier the better)** | **Dani** – “I did, yes. I was a little disappointed, I have to say, and I did point out when I first started the survey that I have had rheumatoid arthritis for many, many years. Nearly 40 years now, and so when I was reading through the book, I just thought, I've heard all this before and it's common sense really. I suppose to a new person, you know, a newly diagnosed person, I think it might have been more helpful. If I'd have had that 40 years ago… you know, I might have saved myself a couple of years of grief.”  **Liz** – “Yeah. Um, also when they first get diagnosed, sometimes it's a lot for patients to take in. And you bombard 'em with all this, and you're like, 'Oh, my goodness.' I can't, I can't take this all - all this in. Yeah, I would probably say six months when they're into it, because then they've got their head round it and they, they're in a routine, then, as to when to take medications and what medication to take, and tried some, and...”  **Mavis** – “Erm, I - well, the sooner the better, really, because they, they - their first biggest impact is employment. If they're already in employment then obviously that's at risk…”  **Niamh** – “And I think, if you'd been, er, sort of, newly diagnosed, I think it would be fantastic. I think it would be really, really good.”  **Tina** – “Yeah. No, I knew probably 50 per cent of it or a bit more than that but I - it was, it was beneficial. I suppose when you're first diagnosed, you read up on everything and anything you can.”  **Willow** – “So I think me, I was hungry for information, you know, as soon as I, as soon as I thought, well, what does this entail? Information on the medication I was on, how my life will change, how I need to adapt. I think to me, the earlier you step in, the better.” |
|  | **Self-Help Book’s Relevance and Usefulness** | **Dani** – “I did, yes. I was a little disappointed, I have to say, and I did point out when I first started the survey that I have had rheumatoid arthritis for many, many years. Nearly 40 years now, and so when I was reading through the book, I just thought, I've heard all this before and it's common sense really.”  **Liz** – “I think I went back - I did go back to it a couple of times, but because I don't climb stairs or anything, um, and it is just the lifting of things, um, basically, I just carry what I can carry, which isn't a lot, but, yeah. I don't think it was helpful because, like I say, at work most of the doors are automatic. Um, the consulting room doors, yeah, they are heavy, but I can manage them. I don't have to climb any stairs. So the only thing I have done is just, um, carry less. Even cups, I can't - not in my left hand, I can't carry 'em.  **Mavis** – “Yes, I read them all when I first got it. It was actually I was, I was reading them in conjunction with the situation I'm in at work…erm, and it made me feel very lucky, actually, that, erm, what is detailed in the, in the leaflets, I've actually experienced.”  **Niamh** – “I did read it when I first got it, um, and to be honest, there wasn't a lot of information in there that was new to me.” |
|  | **Needs support from line managers and colleagues** | **Janet** – “Yeah, so - well, firstly my manager, my peers and obviously all the people that I work with, they know that I've got arthritis and that I do struggle with certain things. So I can often ask anybody if I need to move something or if I need to get something; it's never a problem. I can always say to somebody, you know, 'Can you reach that for me' or, 'Would you mind getting that for me' or particularly if I drop something on the floor, I can't get down. So, and, and, and it's never - it's, it's, you know? Everybody just does it and, and it's - so I never feel uncomfortable…”  **Janice** – “She's [the line manager] very, yeah, she's very supportive and, I mean, she always says that if I feel I need time out, just to take it, you know?”  **Mavis** – “Although I must say, there is an undercurrent, er, in the workplace - whilst on the surface they are being very supportive, there is an undercurrent of, erm, the thought, you know, er, if they're not going to be in work every day, blah, blah, blah, you know…that they need to be managed out or, or not promoted, or - I've seen that many, many times. But on the surface, there's always we - you know, support and, and putting in, erm, you know, adjustments, reasonable adjustments and things like that, but the undercurrent is very different.”  **Mavis** – “Erm, as for looking for job, erm, applications for people who are applying for jobs, it's the employer that needs to be educated. Erm, you know, because if you put an indication on an application form, I've seen, I've seen managers actually skip over and find another reason not to interview…somebody… Yes, I have to say…I've witnessed that and I, I haven't been in a position to actually say…that's wrong because I'm, I'm not at their level. Erm, but I have seen a, you know, oh well, you know, blah, blah, blah. 'Well, what are the qual-, - let's have a look at the qualifications, well, they haven't quite got the…' you know, and they're looking for another reason not to shortlist that person.”  **Niamh** – “I think they need to be more aware of, um, you know, having, having impairments, like arthritis and rheumatoid arthritis, you are dealing with a disabled worker… And, and that you have obligations towards that disabled worker. And then, er, you know, for that to be really, really blatant. And, and then for the information around explaining the impairment and that - how it fluctuates. I think that's one of the key things. Like I said, like, adaptions is - you know, there's some adaptions that can be made…”  **Tina** – “Yeah, they're really good but I think it - I really do think it's dependent on what manager you have.” |
| **Utility of the Booklet** | **Explaining Someone’s Conditions to Others** | **Tina** – “So, and you, you do get fed up with telling everybody the same thing. When you get a new manager you have to go through it all; you're reliving your diagnosis all the time. So those booklets are like, 'Actually, right, look, have a quick read of this or have a quick read of that one.' So for me as well, that was beneficial for what they are.”  **Niamh** – “And get their employer a bit more educated as well, around, um, the parameters of, of basically having arthritis and rheumatoid arthritis. You know, that fluctuation in how you feel and when you're able to, sort of, work, and when you're not able to work.” |
|  | **Making Changes at Work** | **Janice** – “Well, I, I told her [my line manager] I was taking part in it and sort of she, she says to me, 'Is there anything that we could get you that would help you feel better at work?' And at that time, I didn't have a very good chair and she says, 'Well, I'll get on…' 'Cause they have a team, you know, a, a health sort of team. So she sent a request up to the head office asking if I could have a, a chair that was suitable for someone with rheumatoid arthritis. I've got a stool; I've got a stool as well. So, so that if my legs tend to get a bit sore or that, I could put my feet up on the - you know, just a wee stool. So they gave me a stool as well and like the, the pad for leaning on when you're working on your keypad.”  **Niamh** – “Um, I think, um, I did do more of an assessment of my workspace. That's what I did. I did a big assessment of my workspace. Um, I suppose, I suppose, you know that you should do these things, but then whenever you get, um - like, the booklet, sort of, like, prompts you a little bit…”  **Tina** – “And I think having those booklets helped with that 'cause I don't think… It, it gives you, it's almost a toolkit, isn't it, so you can take to your employer. Which is for you and your employer really, so it helps you both. Well, he [the line manager] was better at sorting out - especially 'cause obviously we've been working at home, so at the moment I'm sort of 30 per cent at home and then out in the community and stuff. So it did make me actually think about my setup at home. And about what have I got, because obviously in the office I've had occy health and Access to Work involved so I've got a setup, really good. And a workplace station so I think from doing that and reading all that, it was like: okay, we need to make sure you've got the same set up at home. So that was done and they've bought me a, a right - I've got, they've got me a proper work chair and - which is really heavy so they had to carry that out home. And then they brought it up into my spare room. And that wouldn't have happened, I don't think, without sort of going through things in that and that's, you know, that's, that's really good for being part of this study because that was… And obviously if that was in the last 12/18 months then that probably wouldn't have happened had I not been involved. And that has, that has - it was massive! But I've now, at home I've got two screens and a separate keyboard, a separate…  eg-, egon-, I can't say the word! Egonomic [sic], egonomic mouse. So it's - my hand's, like, level, not twisted. I've got a high screen thing. I've got a special laptop sort of level thing now, and I never had any of that. And my chair, there - I've actually got a work chair in my, in my room so - and anything I want they said, 'Just go…' I have to do the workstation assessments every 12 months and then anything that I need renewing or I, I need anything else to help, then…” |
|  | **Refreshing Information** | **Mavis** – “They are in my bedside cabinet… erm, and have been ever since I had them, and I do have a look at them every now and again. But there's been no occasion where, erm, I've had an issue at work and I'm thinking, well, they should be doing that, they should be…”.  **Tina** – “And then you sort of forget about it; you get on with what you've gotta do to try and manage it that way. So it - for me it was also - and I don't… It's a bit of a refresher about: actually this is what I've got and this is… Don't beat myself up all the time about why I'm so tired all the time or why I'm really hurting because I, I think that's a really important thing that we forget.” |
