# Supplementary material for: WORKWELL process evaluation: qualitative data analyses of the participant interviews at 12- and 36-month follow-ups
Source: Rheumatol Adv Pract. 2025 Mar 14;9(2):rkaf034. doi: 10.1093/rap/rkaf034 (PMC11930348; doi:10.1093/rap/rkaf034)
Supplement: rkaf034_Supplementary_Data [file rkaf034_supplementary_data.zip › Supplementary Table S3 Primary Themes Intervention and Control Groups 12-months (PPI).docx]

Supplementary Table S3 Primary Themes 12-month Follow-Up (PPI)

| **Themes** | **Sub-themes** | **Participant Quotes** |
| --- | --- | --- |
| **Intervention Group** | | |
| **Education** | Acquiring Knowledge | “It was quite a shock to me to be diagnosed. So, I was keen to be involved in anything that was gonna help me, really, and help me stay in work and help me deal with it….It’s been amazing and it helped me stay in work”  Participant V18  “It just put things in front of me that I wouldn’t have ever thought of myself.”  Participant V19  “Lots of …. Resources…so I got a lot out of it that I could use in the future as well as share with others”  Participant V20 |
|  | Practicalities | I” I think I will be working longer because of this…Than I would’ve been had I not been involved in this”  Participant V19  “I was not aware of the equipment I could ask my employer for …. I had the tools that I could then present to my employer and he then provided the equipment that did make my life easier…I have a lot of input on a computer. From half seven in the morning until five o’clock at night…. the ergonomic mouse, that really helped”  Participant V12 |
|  | Confidence | “With the OT, …. who I was assigned to …she give me lots of help and advice, around my condition and work, which also give me the confidence …in what I was doing and what I could …and show my employers …in order to help with work as well. …also, I got a range of exercises and certain things, which also helped …improve and stabilise my condition at certain times.”  Participant V20 |
| Support | Problem solving | “Well, the advice that I got was really good. Problem solving was really good, it really did help me quite a lot, to be honest with you, and I didn’t think it would. But the person that was helping me sort of put things into me and then I sort of worked it out for myself as to what would be best for me.”  Participant V19  “the support and the understanding that I’ve received has been exceptional…I ride a motor bike, and at the time, I really didn’t think I would ever get back on my motor bike…..we worked through that and sort of set a goal of what steps I could do to achieve that”  Participant V18 |
|  | Emotional | “But when I went to see …. the therapist part of it …..I’d sort of put a lot of things and dealt with a lot of things that I didn’t realise I’d put it sort of at the back of my mind…..how my work related to my rheumatisms. …and she sort of…. I’ve got to be open and speak about it which I don’t generally do…. she quickly established a few points that sort of helped me move on.”  Participant V13  “I thought my life had stopped, but actually, it didn’t. And the …Workwell with, my occupational therapist helped me get it in perspective and set …reasonable goals “  Participant V18 |
|  | Help and advice | “It just put things in front of me that I wouldn’t have ever thought of myself. I’ve got arthritis in my hands, my fingers, and I struggle squeezing colour tubes out. The (OT)…. wonder if you could get something that could squeeze the tube……I got a tube squeezer…I can’t tell you what a help it was……I’ve now got ….ergonomic scissors…..that I didn’t k now existed. To save my shoulders and my elbows and my arms lifting up.  Participant V19 |
| …Motivation | Empowerment/  Enabling | “I’m really interested in research; I’m really interested in the future and helping…. I’ve had a tricky time at work and it’s definitely ..helped me through…… the difficulties as well as….concerning ..my confidence in certain things in terms of work as well”  Participant V20  “The employment I was at, I’ve got no back-up (*from*) Management. …. But because of Workwell I actually decided…and said right okay, I’ll make a change. So, I did make a change of occupation. …it blatantly shown me what the issue was…. I hadn’t been accepting and I’ve just, sort of, carried on and on……pinpointed by an expert….I’d been more open than I would ever be because I tend to keep things to myself. But I decided to say, right, okay fair enough I’ll act on this.”  Participant V13 |
|  | Knowing your disease | “I thought it would be a good idea to participate because the more knowledge we get about it and how it relates to work, and life is…the better chance we’ve got with understanding the disease.”  Participant V13  “Extra information…then basically we can challenge our daily activity....and carry on with life as best we can as normal”  Participant V12 |
| **Control Group** | | |
| Motivation | Helping others | “to help people that are trying to stay into work that have got, um, deb, deb, debilitating disease**.**” V03  “it made me feel like you’re, you’re, you know, you, you’re making things better for other people.” V03  “it might help people in the future.” V07  “you need people to do these things, to help, you know, help with it.” V08  “my input might be useful.” V14  “I just wanted to, uh, help other people and myself” V15  “I’m prepared to help anybody who’s in the same situation as I am.” V21 |
|  | Informing sharing | “I would hope that my contribution, um, you know, informed the researchers, um, with information that could actually be useful for other people” V06 |
|  | Knowledge | “I thought I might find out something” V10  "increased my awareness… more confident…. It was nice to get phone calls and have…. Human contact" V11 |
| Symptomology | General | “and I think if I hadn't have done it, I think, I wouldn't have thought as much as I did about, you know, pain, working, um, and, and all those things that it sort of covers, um, I wouldn't of really thought that … I wouldn't have given it much thought, I don't think” V07  “I did, I did also find that, um, there's things that I have done that I wouldn't have thought of doing, um, if I hadn't done the study”V07  “it allowed me, in a selfish way, to reflect on actually how I was feeling, um, because I think we get so wrapped you in everyday life, it gave me that time to think about where I was actually at with my journey.” V09  “it kind of got me thinking about what I was doing and when and how.” V15  “made me think about my lifestyle and how, how I go about things basically.” V15 |
|  | Employment | “made me a bit more confident, perhaps, is the word to, like things with my employer to say, you know, I need to change my working pattern or things like that and, and feel like it was an okay thing to do” V11  “ I think being s … s … self-employed erm … some of the things could have been more, eh, directed, erm, to the fact of, erm … m … more having to … possibly having to go to work than, erm, not going to work and not getting paid for it basically” V21 |
|  | Acceptance | “… I hadn’t, I hadn’t been accepting” V13 |
| Accessibility |  | “I never struggled with it at all. Yeah. It was, it was clear” V08  “I think the questionnaires were easy to understand, easy to fill in, easy to return” V09  “it was all right, I could understand it” V10  **.**  “I found it quite easy to read” V11  “it was all, all really, really quite, quite clear” V15  “The information was very clear, yes. Erm, no problems at all understanding it” V21 |
