# Supplementary material for: WORKWELL process evaluation: qualitative data analyses of the participant interviews at 12- and 36-month follow-ups
Source: Rheumatol Adv Pract. 2025 Mar 14;9(2):rkaf034. doi: 10.1093/rap/rkaf034 (PMC11930348; doi:10.1093/rap/rkaf034)
Supplement: rkaf034_Supplementary_Data [file rkaf034_supplementary_data.zip › Supplementary Table S4 Primary Themes Intervention and Control Group 36-month Follow-Up (Researcher).docx]

Supplementary Table S4 Primary Themes 35-month Follow-Up (Researchers)

| **Themes** | **Sub-Themes** | **Quotes** |
| --- | --- | --- |
| **Intervention Group** | | |
| **Long-Lasting Impact of the Trial** | **Positive Changes at Work** | **Amanda** – “Oh yes, I, I, changed the car I'm driving. Now I, I do get a Motability car which is much easier to drive in. So it's much more comfortable, it's automatic, so when my leg was physically bad, it was, erm, it's stopped that, stopped any extra pain using a manual […] I, I had a different desk, one that was adjustable, with a different seating. I had a, a laptop riser, so it's easier to see.”  H**ayley** – “Well, the main, the main things really were, er, about communication and sort of, erm, changing the types, you know, making sure that I wasn't doing repetitive work, and so changing tasks that I was doing to, to move about and that kind of thing.”  **Jemma** – “Yeah, yeah. Well, I, I mean, when I've had a flare-up, um, yeah, it has helped. Um, and what else actually work has done for me, um, is that I get changed, um, on w-, where I first go in the building. Um, they've got me a changing room there, so I don't have to use the stairs.”  **Pam** – “Absolutely. Erm, completely from the, the handbook and the information that I received to, erm, the one-on-one sessions I had with the OT, erm, absolutely, and, erm, have continued using that, erm, up to this day.- […] So, erm, ensure I get regular breaks. Erm, not carrying heavy loads. So, using, erm, a wheeled kind of case or a wheeled bag, erm, so that I'm not carrying, erm, really heavy things. Erm, I do a lot on the computer....and, erm, needed adjustments in terms of risk supports, a different sort of mouse, erm, what else did we do? And, erm, some finger splints, and all of those things I continue exactly as I did then.”  **Rose** – “Yeah, it was specific about, um, about measuring my time and my speed, and, um, you know, so I could only have like five spoons in a day, and, um, I had to allocate what those spoons were, and once the spoons were used, I couldn't use them again. Um, and so, if I was doing like big jobs where I was maybe sat for a long time, um, at work, or even in my private life ... and then when I've used the five ... it was really - it was things like that, it was really good advice. Yeah, so I would set myself alarms on my mobile phone, um, so if I said I was going to work on something for an hour or an hour-and-a-half, that was one of my spoons, I would set my phone, and, um, when the alarm went off I knew I'd run out of time, and I had to sort of - I had to try and stop and come back to it tomorrow, things like that. Also, just remembering because - well, it was great to work from home, but what I find is that you tend to just sit and work. You don't have those interruptions, nice interruptions you have in a workplace.” |
|  | **Empowered to Advocate** | **Amanda** – “Yes, yes, I could speak to a line manager and help me.”  **Hayley** – “Well, I mean, I'm, I'm quite confident, you know. I mean, this, I, I've done, you know, I'm significantly more intelligent than my job, job suggests, without being silly. You know, so I, I do think that I've got the capacity and the skills to be able to talk to people about this.”  **Jemma** – “Yeah, I always was always wary of saying, you know, 'I can't do this call, I can't do that,' because I felt I would be, erm, bypassed for any other calls .. So, as I say, I'm actually taking more time off sick now or because of issues.”  **Leanne** – “It really did help me because I didn't realise just how much I was entitled […] Because there might come a day when I really do need it, so there have been occasions with the situation I've been in recently where I've just thought, do you know, I'll just go off sick and have a break and - because I can't deal with this.  **Pam** – “Erm, no, absolutely. Erm, I think, erm, I, I think the, the being on the programme definitely gave me those extra sort of hints and tips and tricks of what I can do, and I think the confidence to perhaps seek support through my employer. Erm, I may not in the past have actually said to them, 'I want an ergonomic assessment,' erm, and struggled on. Erm, but, certainly, I think being part of the programme gave me the confidence to say upfront, 'I want an ergonomic assessment, and these are the reasons why, and this is the, the, the small amount of adaptations I need.” |
|  | **Positive Experience of being involved in the study** | **Amanda** – “it has helped me, helped me to stay working, yes.”  **Joanie** – “No, I don't think so. I did get some, you know, support from, I think, it was the rheumatologist, not the rheumatologist, the OT at the time. She did help me out quite a bit […] I think it was all quite relevant really.”  **Leanne** – “Just to say thank you to the team that did support me […] I could tell that she'd [the OT] put a lot of hours in, so I was really grateful. It was like really nice to receive everything.”  **Pam** – “Erm, no, I just thought it's been amazingly well, so amazingly well, erm, you know, organised and I've loved receiving the ongoing emails, erm, even though they're quite, erm, widely spaced apart, it's lovely to receive those emails and kind of hear, oh, great, the study's still going on and I really appreciate the fact that you still wanted to talk to me even though I'm, erm, a little bit further away. Erm, I think.”  **Rose** – “I thought it was excellent. I thought it was really good. Um, I thought I was very fortunate at the time. Obviously, I was, I was asked if I would do it when I was first diagnosed, and I filled out the forms, and then, of course, the trial was stopped because of COVID, but then, obviously, I got on it again, and I was really, I was really pleased to do that. Yes, I was very fortunate to do that, and I can tell, when I am doing - so even when I was doing the fatigue sessions, some of the things I was talking about in that session of my coping strategies, um, my occupational therapist would say to the rest of the group, 'Oh, Fiona's done - she's on the Workwell trial,' um, because she was recognising - there was another occupational therapist there as well, and they were recognising some of the - my wording and my techniques […] I think it, um, it was fantastic, and, um, you know, if anything … the more, the more ... the research and the element of both medication and also the techniques, I think it's, I think it's invaluable, because, um, it's one of those hidden things, isn't it, that people don't realise how debilitating it is.” |
|  | **Understanding the Importance of Self-Care** | **Joanie** – “But it was - a lot of it was about making sure take time for myself, you know, to make sure …that I'm functioning the best I can […] It was worthwhile doing, it was definitely worthwhile doing and it did make me realise how to look after myself.”  **Leanne** – “No, not at all. I think it went over and above. I think it was absolutely outstanding. I don't, I don't think there was anything more that I could have got. I think I even got emotional support. I got, erm, advice about mindfulness and, erm… You know, being, being a bit more healthy for myself.”  **Pam** – “Yeah, absolutely. I think it's, erm, I would say, as healthcare professionals, we're very good at looking after ourselves and I think being part of the trial reinforced to me that for me to be the best at what I do, I have to self-care. So, erm, absolutely, erm, it's been invaluable. Erm, I think what the study did was make me more aware of taking regular breaks and making sure I have good-quality rest.” |
| **Long-Term Barriers and Enablers to Workwell Implementation** | **Recalling Information** | **Jemma** – “Uh, yeah. They [sigh] advised me to, um, because my lunch, uh, room, where we, where we were told to go and have lunch, um, is upstairs. So, they did advise me to maybe ask about, um, asking if there's anywhere, like, on our level, where we work.”  **Joanie** – “At the minute my brain fog is, is absolutely atrocious. But it was - a lot of it was about making sure take time for myself, you know, to make sure that I'm functioning the best I can. But [I can] not particularly [remember] a lot of it.”  **Joseph** – “Not really, no [can remember what I did].”  **Pam** – “Absolutely. Erm, completely from the, the handbook and the information that I received to, erm, the one-on-one sessions I had with the OT, erm, absolutely, and, erm, have continued using that, erm, up to this day.”  **Rose** – “I did. Um, I did, but I found that, um, it was, at the time when you were doing it, and you're fully embedded in it, um, you were, you were a bit like a talking, um, phrasebook, um, advice book, but as the time's gone on it starts to wane a bit, and then you start to doubt yourself and, um, I think - but my, my mood was very low when I was first diagnosed, and then it helped sort of build it up. My mood recently has been dipping again, because I think that it's that gap, it's that not being involved in anything, and feeling a bit forgotten about” |
|  | **Relevance** | **Hayley** – “So a lot of the things that were suggested to me were things that I was doing almost naturally, um...”  **Pam** – “Yes. No, no, no, I just think the whole study is brilliant. Erm, erm, I know there's such, there's such little support out there for - I mean, I've got psoriatic arthritis, but there's such little support out there and such little kind of, I think, erm, focus on trying to keep people in the workplace as much as they possibly can, erm, and I think that's so important when you've got any chronic condition, erm, to kind of feel that you are useful and you can maintain your job or your role or that kind of thing. So, I, I think the project's invaluable and I hope, whatever the outcomes or the primary outcomes or whatever you've come up with, erm, I hope that it really spurs on clinical practice and what happens for people in the future.”  **Phoebe** – “I didn't really receive an awful lot of advice…”  **Rose** – “Well, I'm not really sure, because, obviously, it was, it was a trial programme, and, and I knew it was [unclear words 0:07:52.7] to an end, it wasn't going to last forever, but I think I would hope, moving forward, that there would be like, er, maybe not such a strong focus, but like a, a continued, um, attachment.” |
|  | **Receptiveness of the Work Environment** | **Jemma** – “Um, I've got a really good senior, which is really good. Um, and, to be honest, she, she suffers, um, with her, with arthritis as well. So, she, we've got quite a thing in common. So, yeah. So, she kind of understands, um, where I'm coming from. And I have disabled badge as well, that I have. How, is it, it's two years, isn't it?”  **Joseph** – “There were times where there's, like, some supervisors, new ones, erm, they'll say like, 'Oh, he's limited in what he can do,' and they'll turn around and doubt us […]. There's nothing on us as you can see, you know what I mean? Because it's an invisible disability. You can't look... Like, look at a person's back and go, 'Ah, you look, you look fit enough for me.' But obviously I'm not.”  **Pam** – “Erm, again, when I was in the study, I had over an hour's commute to and from work, erm, and it's exactly the same now. Erm, so I don't think the study could have changed that for me, erm, because there wasn't an option for me to do what I was doing from where I was living at the time, and the same is now until we get to work from home. Erm, I think what the study did was make me more aware of taking regular breaks and making sure I have good-quality rest.”  **Phoebe** – “And I think my employers are a lot more understanding than my previous employers as well, so…” |
| **Control Group** | | |
| **Developing Self-Awareness** | **Questionnaires as Instrument for Reflection** | **Andrew** – “Er, yeah. Er, it, it [completing the questionnaires] kind of puts into perspective where you are, and it, it, it helps you refocus as well.”  **Karen** – “Well, I q-, I like the way that you can record if things are changing or not. I like the questionnaires because, yeah, because you can, you've kind of got like a, you've got, you've got, like, um, you can, um, see if anything's changing in the arthritis itself. So I quite like the questionnaires. Yes, it's kind of like a reminder. You're just kind of paying attention to what's happening? Because then, you know, because you could've had a higher or a lower score on the previous questionnaire and it, you can kind of track it.”  **Diane** – “Um, in, in terms of the study, I think - I sort of always felt, as I was filling in the questionnaires, that I wondered if I was the sort of candidate that you, that you needed for your study [chuckles], um, because I don't feel that I've had to make a - or I didn't feel, initially, that I'd had to make a lot of adjustments for my rheumatoid arthritis, but actually, what I realised as I was answering the questions, is that the job I do gives me an enormous amount of flexibility that maybe other people who are employed don't enjoy. So in terms of how I set my environment up, and particularly in terms of the number of hours I work, and, um, I, I can, up to a point at least, um, choose. So if I'm having a, a not-so-good day, I am free to cancel lessons, and my pupils understand that, you know, if I say I'm not well, then I'm not well. And, um, yeah, so I think, I think, really, for me, that's what the study did; it, it actually made me feel very grateful that I'm in this situation with this kind of flexibility, yeah. Yeah. And actually, when I - it, it reminds me, when I first got diagnosed, the hospital used to ask me to score myself. They used to say, 'How are you feeling today?' and I can't remember - was it out of 100, maybe? - and they used to say, you know, 'What, what number would you give yourself today?' and I used to just think, huh, but actually, it just made me stop and think, well, how do I feel? You know, because life is so busy, isn't it? You know, and, and, and it just made me stop and reflect, and think, yeah, how do I actually feel? Oh, I feel like I'm a 70, or, you know, or whatever. Oh, okay, yeah, I feel okay. That's good. You know, and so, really, that's what the study did; it kind of threw things up that maybe I'd not thought of or not given myself time to think of, or had quietly thought, oh, well, it's not happening to me, so I won't think about it, and in answering the questions, it made me just stop and think, and be more aware, oh, yes, this condition can cause other problems that I'm not experiencing, I should at least be aware of that, and just, yeah, reflect on things. And, and, er, and, and I feel now, I think going back to what you originally asked, I do now feel a confidence, that, yeah, I made the right choice there. Um, I know the hospital are definitely happy that I didn't go back to teaching, but I feel confident that, yeah, I made a good choice to stay in this job, because of the flexibility and the longevity and, er, yeah, I feel good about that. So thank you.” |
|  | **Changes due to the Booklet** | **Joy** - So if I wasn't feeling great, I was, you know, I was able to, err, to rest up a little bit, which, um, is all, you know, in the pack, isn't it? It's trying to help yourself, yes, and li-, listen to my body I guess, instead of ignoring it.”  **Andrew** – “Yeah. Um, yes, it has, it has helped. I've learnt a little bit more about how to pace myself and, er, er, as a result getting... Probably getting a bit more done than I would have done if I'd tried to, er, just go like a bull in a China shop at it to get it finished.”  **Margaret** – “Yeah, well, it kinda encouraged me [the booklet] to speak to my manager about how I was feeling. Which is something that I would probably have tried to hide before and not go on about, you know.”  **Diane** – “Yeah, so for me, it just gave me a huge amount of awareness. I maybe didn't, didn't change anything, but, you know, knowledge is power, as they say, so just to be aware that, you know, this, this condition could give me other sorts of difficulties, and just to be aware of that, and just to kind of have a glance at my working environment and the sorts of things that I do. Yeah, I think that was, that was really useful for me.” |
| **Limitations of the Trial** | **Struggling to remembering the booklet** | **Iain** – “Yeah, I think so roughly [about remembering the booklet], yeah, yeah.”  **Grace** – “I don't think I've got that self-help... Have I? If I have, I haven't read it. Sorry.”  **Linda** – “o be honest, I would have to be prompted to see if I had or not because my memory has just, erm… It, it's dwindled over the last couple of years. I'm actually going through the menopause…and I've got what you call foggy brain.”  **Brenda** – “Er, I'd have to, I'd have to go back and, and look through it again?” |
|  | **Not finding relevant information in the booklet** | **Iain** – “Yes, I did have a look at them - but I didn't find… To be truthful, I didn't find much help in them. R: Okay, so what was the type of help that you were looking for that wasn't in the booklets if you - do you remember? Well, to be truthful I, I, I, I think it was just to… It was something that I thought that maybe I could've - that would've helped me to keep on working. I didn't really want [chuckles] to retire. I don't think most people do but… Well, eventually I - it, it, it just go too bad that I just couldn't… Well, I just couldn't do my job so…” |
|  | **Need of Upward strategies** | **Karen** – “It would, it would be good if there was more help, not, not from, like, you guys but, like, government help from a, uh, knowing where to go kind of situation.” |
|  | Asking help outside the trial | **Joy** – “Yes, it was. Yes, yeah. Yeah, yeah. On top of, um, the pack I had a lot of counselling, as well.”  **Brenda** – “But, erm, I had a fantastic consultant in the early days who had a really positive mindset. Erm, and she - so I was a PE teacher back in the day. And she was the one that said to me, 'Go golfing, go skiing. It might mean that you have to spend a day in bed afterwards or whatnot, but so what,' you know. And that was a really, erm… that was really helpful to me.”  **Karen** – “Um, and n-, not really, um, the, the advice is, it's, it's, well, yes and no. It's like it's coupled along with advice from my union and Citizens Advice and things because I had some problems at work, but it's all kind of, all together it's helped […] Yeah, I have an occupational therapist already and she's really good, bless her heart, so I've got a chair in my bathroom now, I've got a chair in my shower, but I, I don't know what I need.”  **Diane** – “Oh, sorry. I was just going to say, I think, I think what works really well for me in terms - and I don't know if I can sort of link this to what you're saying there about that booklet - what works very well for me in my management of my arthritis is, at the hospital, um, er, you know, where my consultant is, there's a phone line they have for, um, for patients with my condition. Probably other conditions, as well, but it's - and it's somewhere I can go to if I've got a question, and maybe in the course of 12 months I might phone them once, twice, you know, and it, it might be, 'Oh, I've got this, um...' So, you know, I remember phoning them about I'd, I'd got the - I don't know, maybe it was something like the flu vaccine, and, 'Oh, do I take my medication when I've, you know, when I've got this vaccine? What do I do.' Um, and actually I really appreciate just knowing that they're there, in case I've got questions. So just going back to that booklet, which might be a sort of cheaper option than what I'm going to suggest, but, um, I suppose the reason I said I wished it was the other, the other option, was just what I value so much is thinking that there might be somebody there if I've got a question. And I might, I might very infrequently have a question, but it's just knowing, oh, there's a human being that I can just ask this question to. So that's kind of why I was desperately hoping for the other option, because I felt, for me personally, that's, that's what would have worked better for me, but maybe that would be a more expensive thing, rather than sending people information, you know, in a pamphlet or booklet.”  **Helen** – “Um, probably not, because to be quite honest, um, where I work, they're very, very good with, um, anybody with a disability, they'll make adjustments at work. Um, I can work from home if I need to if I'm having a bad day, that… So, yeah, they're very good.”  **Judith** – “…as soon as I was diagnosed, I delved into everything I, you know, on, on the web, the websites, er, you know, source of information and, and, you know, and the rheumatology nurse that was assigned, she was helpful too. All that sort of stuff. But I suppose people who were newly diagnosed and I actually, actually, I've passed that information on to another member of staff who is newly diagnosed with rheumatoid arthritis and was struggling, and that was a young person, a male person, actually. And I passed, erm, the information on to him, er, to help him sort of cope…” |
